# The 4E-BPs as Translational Regulators in Neurological Disorders: Molecular Mechanisms and Therapeutic Potential

**DOI:** 10.1007/s12035-025-05553-6

**Published:** 2025-11-24

**Authors:** Sindhu S. Baskarapantula, Venkata Surya Kumar, Priyajit Changdar, Debashree Chakraborty, Yogendra Nayak, Albert R. La Spada, Craig L. Bennett, Somasish G. Dastidar

**Affiliations:** 1 Center for Molecular Neurosciences, Kasturba Medical College, Manipal, Manipal Academy of Higher Education, Manipal, Karnataka 576104 India; 2 Biophysical and Computational Laboratory, Department of Chemistry, National Institute of Technology Karnataka, Surathkal, Mangalore, 575025 India; 3 Department of Pharmacology, Manipal College of Pharmaceutical Sciences, Manipal Academy of Higher Education, Manipal, Karnataka 576104 India; 4 Department of Pathology and Laboratory Medicine, University of California, Irvine, CA 92617 USA

**Keywords:** 4E-BP1, 4E-BP2, 4E-BP3, Protein translation, Neurodegeneration, Neurodevelopmental disorders

## Abstract

Protein translation is essential for maintaining the optimal functioning of the human nervous system. The 4E-Binding proteins (4E-BPs) are central regulators of this process, acting on the initiation factor eIF4E. Three homologs 4E-BP1, 4E-BP2, and 4E-BP3, are differentially expressed, with the phosphorylation state controlling cap-dependent translation in response to diverse physiological inputs, including growth factors, cytokines, hormones, nutrient availability, and signaling cascades converging on kinases such as mTOR. Dysregulation of 4E-BP activity has been implicated in multiple disease contexts, including neurodegenerative disorders (e.g., Parkinson’s disease, Alzheimer’s disease), neurodevelopmental disorders (e.g., Autism spectrum disorder, Epilepsy), neuropsychiatric conditions (e.g., Depression, Schizophrenia), and autoimmune diseases (e.g., Multiple sclerosis, Guillain–Barré syndrome). We aim to explore the importance of 4E-BPs through a neurological perspective and understand their role as therapeutic targets. In this review, we provide a comprehensive analysis of the three 4E-BP homologs in the central nervous system, emphasizing the CNS-specific dominance of 4E-BP2 and its link to synaptic plasticity and cognitive function. We further examine and provide mechanistic insights into how 4E-BPs contribute to disease pathogenesis, highlighting both shared and disorder-specific features. Finally, we discuss current therapeutic strategies aimed at modulating the mTOR/4E-BP axis, outline the limitations of existing approaches and identify emerging avenues for drug development. Together, these perspectives underscore the potential of 4E-BPs as both therapeutic targets and biomarkers in neurological disease.

## Pathways Related to 4E-BP’s Function

### The mTOR Pathway

**Fig. 1:**
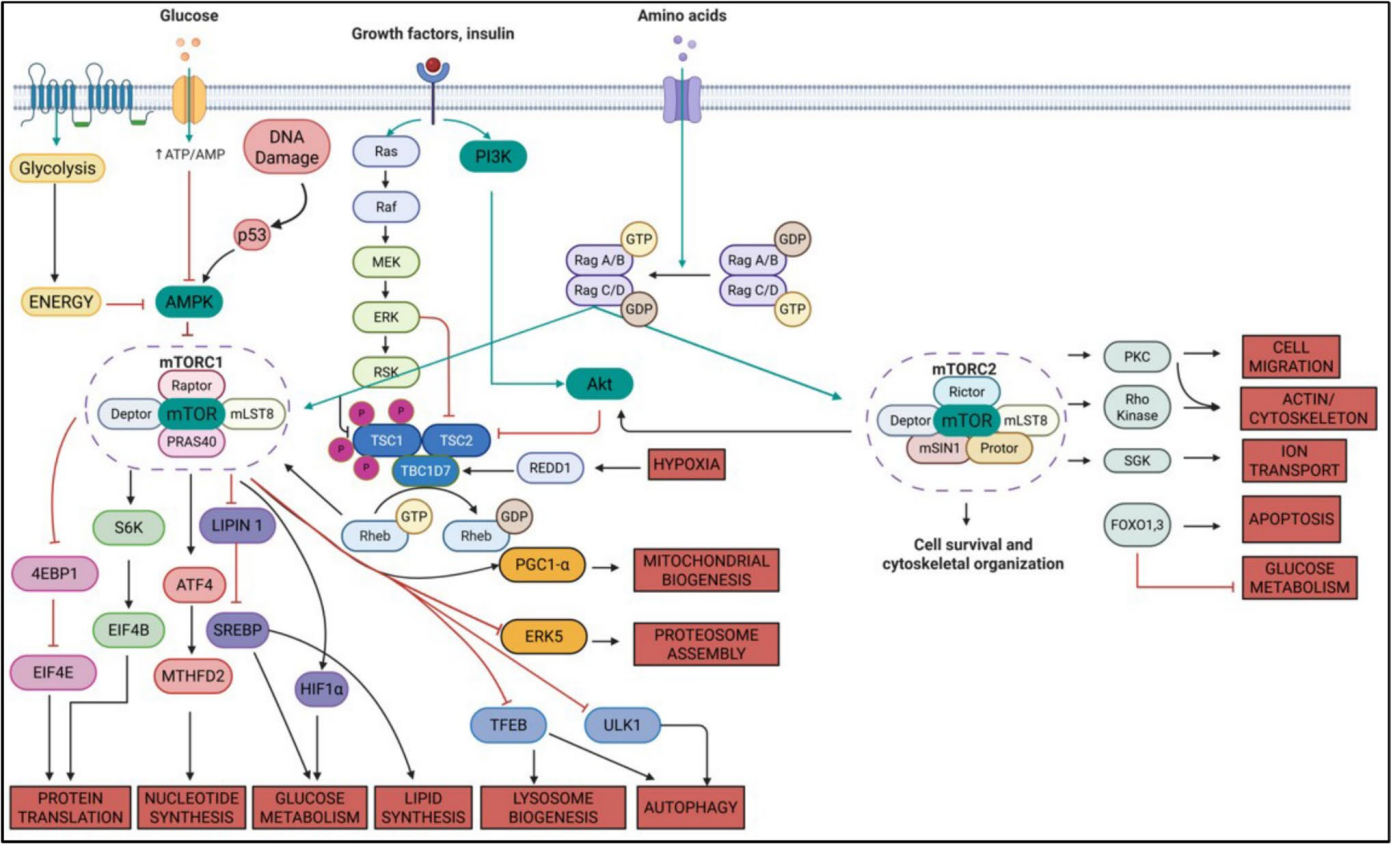
Schematic representation of the mTOR signaling pathway and its downstream targets. The mammalian target of rapamycin (mTOR) pathway modulates cellular homeostasis and metabolism by combining signals from the levels of energy, growth factors, nutrients, and stress. Activation of AMPK through low energy levels inhibits the activity of mTORC1; on the other hand, signaling through the Ras-PI3K-AKT pathway activates mTORC1. The localization of mTORC1 to the lysosome for activation occurs through Rag GTPases sensing of nutrient availability. mTORC1 controls protein translation, nucleotide synthesis, glucose metabolism, lipid synthesis, and responses to hypoxia. mTORC2 directly affects the organization of the cytoskeleton, ion transport, and glucose metabolism. Both mTORC1 and mTORC2 control cellular outcomes to maintain function and adapt to environmental changes

Mammalian target of rapamycin (mTOR) is a serine/threonine kinase that plays a central role in transferring signals across highly interconnected signalling pathways. mTOR is also known by several other names based upon the initial phases of mTOR research, such as FRAP (FKBP12 rapamycin-associated protein), SEP (sirolimus effector protein), RAPT1 (rapamycin target 1) and RAFT1 (rapamycin and FKBP12 target) [1]. The target of rapamycin, commonly referred to as TOR, is typically preceded by a prefix indicating the species from which it is derived, such as mTOR for the mammalian version or dTOR for the *Drosophila* version. Rapamycin was first extracted from *Streptomyces hygroscopicus* found in the soils from Rapa Nui Island by Surendra Nath Sehgal, and his work was published in 1975. Rapamycin was first known as a moderately effective antifungal compound. Later, it was identified as an effective anti-neoplastic agent and an immunosuppressant [2]. mTOR is a master regulator of different pathways, such as glucose metabolism, protein biosynthesis, lipid biosynthesis, nucleotide biosynthesis, mitochondria and lysosome biogenesis, cell cycle, differentiation, hypoxia, and autophagy [3] (Fig. 1). It is also interrelated with other pathways, such as the phosphatidylinositol 3-kinase (PI3K)/RAC-alpha serine/threonine-protein kinase or Protein Kinase B (AKT), AMP activated protein kinase (AMPK), and Mitogen activated protein kinase/ extracellular signal-regulated kinase (MAPK/ERK) pathways, whereby the interplay between these pathways ensures a balanced cellular response to nutrient availability, energy status, and growth signals [4].

**Fig. 2:**
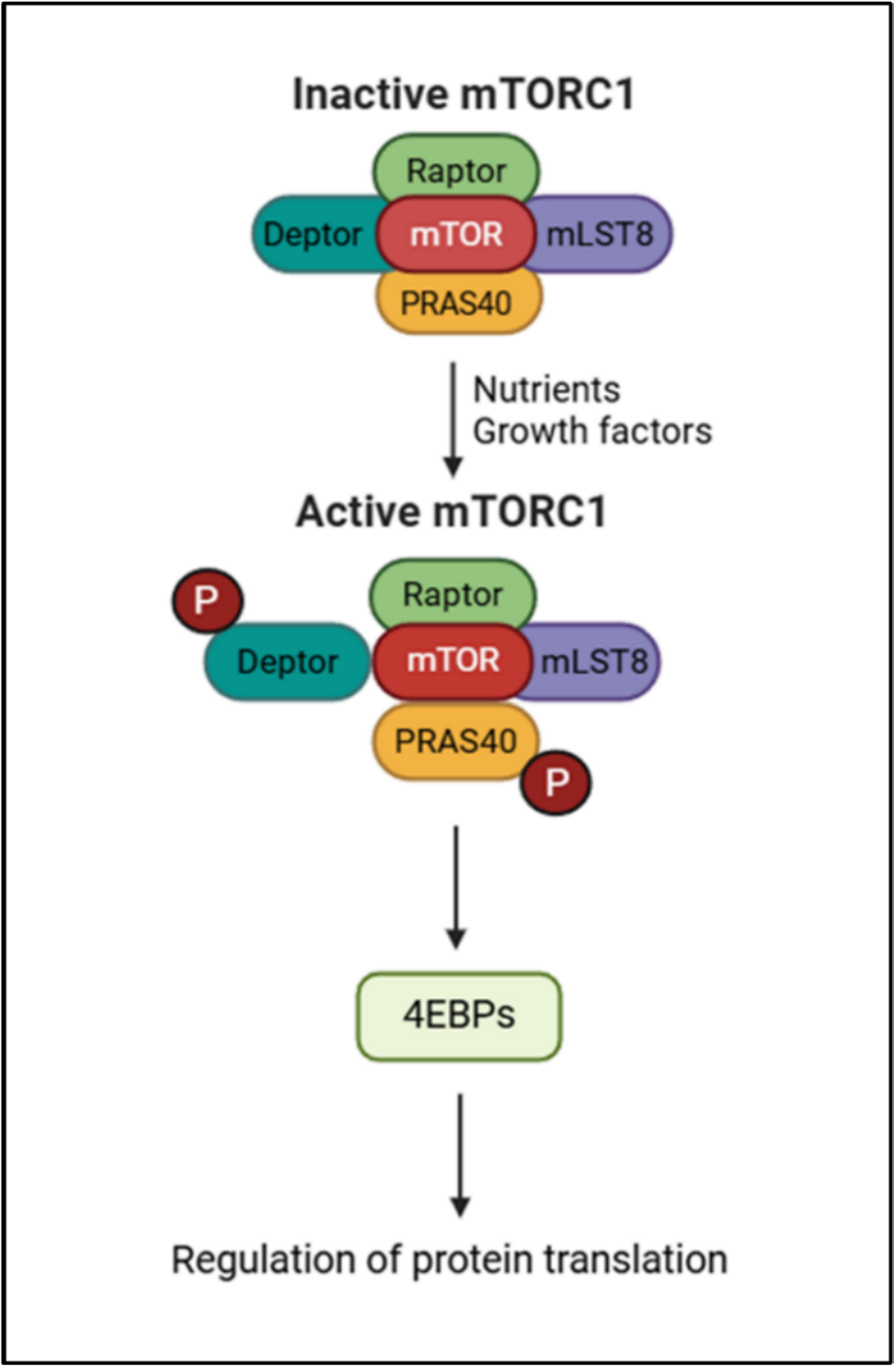
Activation of mTORC1. mTORC1 is activated by nutrients and growth factors by phosphorylating Deptor and PRAS40. This weakens their binding with mTOR, in turn activating the mTORC1 complex. mLST8 and Raptor do not participate in the activation of the complex. Activated mTORC1 helps in regulating its downstream target 4E-BP1

Protein metabolism is tightly regulated through the control of protein synthesis via mRNA translation and protein degradation via autophagy or proteasomal degradation. Such regulation is highly conserved across eukaryotes (humans, fungi, *C. elegans, Drosophila*, etc.) [5]. The mTOR pathway consists of two protein complexes, mTORC1 and mTORC2. mTORC1 is sensitive to rapamycin, whereas mTORC2 is not. Both complexes consist of the core elements of mTOR, DEP domain containing mTOR interacting protein (DEPTOR), Proline-rich Akt substrate of 40 kDa (PRAS40), and Mammalian lethal with Sec13 protein 8 (mLST8). mTORC1 additionally includes Regulatory associated protein of mTOR (Raptor), and mTORC2 includes Rapamycin insensitive companion of mammalian target of rapamycin (Rictor). mTOR is the main catalytic subunit of the complex. Regulatory-associated protein of mTOR (Raptor) helps in complex formation and identification of substrates, whereas Rictor performs a similar function in mTORC2. The function of mLST8 is still unclear. Proline-rich AKT substrate 40 kDa (PRAS40) and DEP-domain-containing mTOR-interacting protein (Deptor) act as negative regulators of the mTORC1 complex. The mTORC2 complex also utilizes nonessential proteins such as Protor and stress-activated protein kinase-interacting protein 1 (SIN1). Protor, known as a protein associated with Rictor, helps in regulating substrates such as Akt and serum and glucocorticoid-induced kinase 1 (SGK1). SIN1 helps in the assembly of the mTORC2 complex and binds to a few substrates, such as Akt. mTORC1 regulates protein translation through its downstream target 4E-BP1. mTORC1 is activated in the presence of nutrients such as glucose and amino acids, growth factors such as IGF-1, and the cellular levels of ATP. When mTORC1 is activated, it phosphorylates PRAS40 and Deptor, causing a decrease in their interaction with mTOR, in turn further activating mTORC1 [6–9] (Fig. 2).

mTORC2 does not directly regulate 4E-BP1, 4E-BP2 or 4E-BP3. However, mTORC2 phosphorylates Akt, which in turn activates mTORC1. Akt is a serine-threonine kinase that is essential for numerous functions of the cell. Akt is activated when it is phosphorylated by PDK1 and mTORC2. Akt then phosphorylates TSC2 (Tuberous Sclerosis Complex 2), leading to inactivation of the TSC1/2 complex. This causes Rheb, a component of mTORC1, to stop converting from Rheb GTP to Rheb GDP. Rheb, in its active form, causes the activation of the mTORC1 complex. Once mTORC1 is activated, it phosphorylates 4E-BPs. Phosphorylated 4E-BPs cannot bind to eukaryotic translation initiation factor 4E (eIF4E), leading to the continuation of protein translation. This is an indirect mechanism by which mTORC2 activates mTORC1 through the Akt pathway. In this review, we focus on mTORC1 and its direct regulation of protein translation through the regulation of 4E-BPs [10–13].

### Mechanisms of Translation Initiation and Ribosome Recycling

#### Protein Translation Initiation in Eukaryotes

Protein translation initiation is a highly regulated, rate-limiting step of protein synthesis. It involves the recruitment of a ribosome to the mRNA, marking the initiation of the process. This can take place either by a cap-dependent or cap-independent process. However, the cell primarily employs the cap-dependent mechanism. The ribosome binds at the 5’ cap (m^7^GpppN, where N can be any nucleotide) end of the mRNA, which is aided by different initiation factors [14]. The most important type of initiation factor is the eukaryotic translation initiation factor 4F (eIF4F) complex, which comprises eukaryotic translation initiation factor 4A (eIF4A), eIF4E, and eukaryotic translation initiation factor 4G (eIF4G). The cap-independent mechanism occurs when the ribosome binds to an internal ribosome entry site (IRES) rather than the 5’-cap. IRES is present on the 5’-UTR of the mRNA, which can bind to the ribosome and initiate translation without the need for many initiation factors or the 5’-cap. This is preferred mainly when the cell is under stress or if cap-dependent translation proteins are unavailable [15]. The 4E-BPs act as key regulators of cap-dependent translation by binding to eIF4E and preventing its interaction with eIF4G, thereby inhibiting the formation of the eIF4F complex [5]. The canonical initiation of protein translation occurs at many different stages, which are explained in detail below.

#### Ribosome Recycling and the Formation of 43S Preinitiation Complexes

**Fig. 3:**
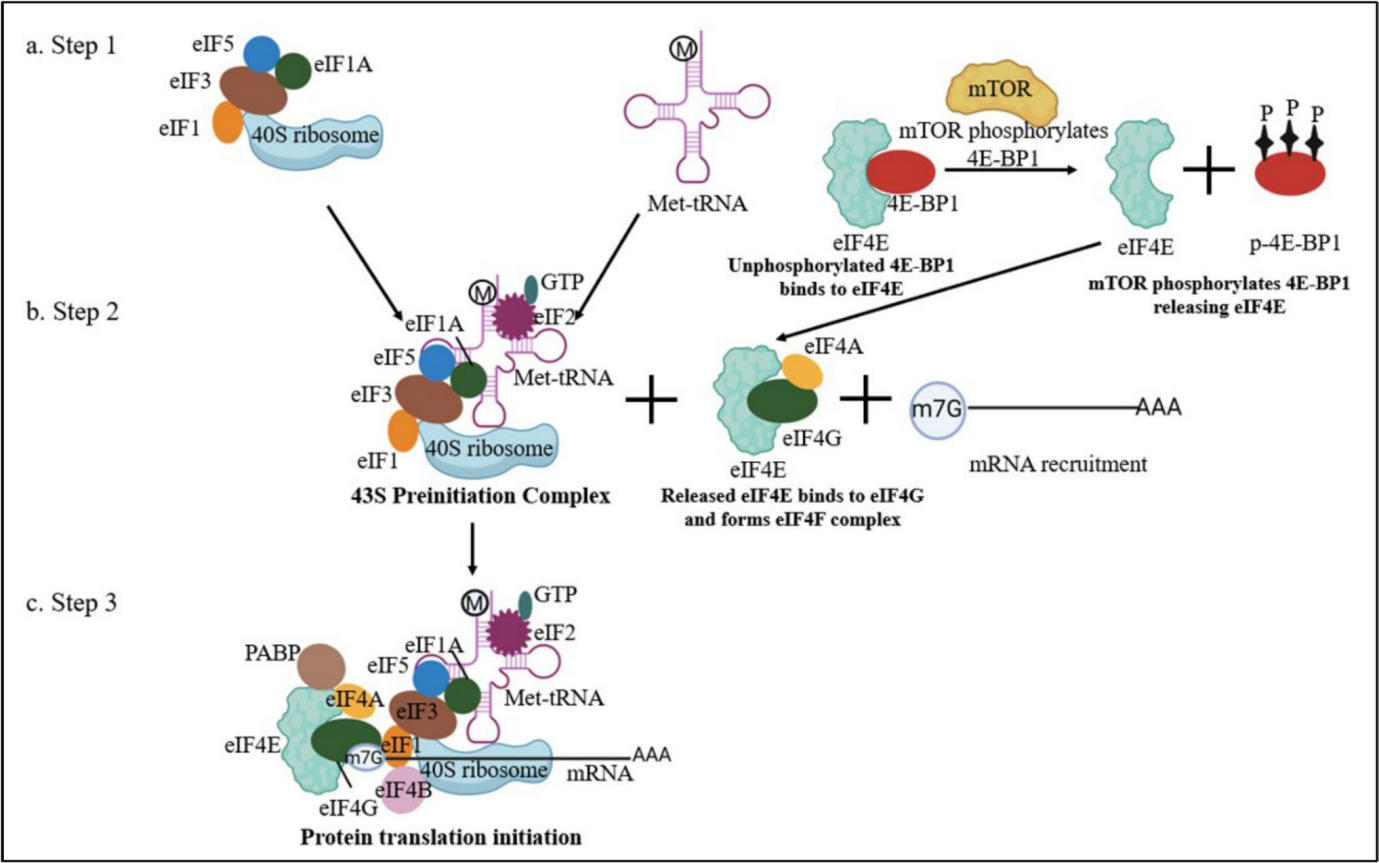
Illustration of eukaryotic protein translation initiation. **a**. Step 1: Briefly, after each cycle of protein translation, the ribosomal machinery and the initiation factors are usually recycled and reused. The 40S ribosome remains attached to eIF1, eIF1A, eIF3, and eIF5, which later associate with tRNA, marking the start of the initiation step of protein translation. Simultaneously, 4E-BP1 regulates the release of cap-binding protein eIF4E. This is a rate-limiting step in the initiation of protein translation. When 4E-BP1 is phosphorylated by mTOR, it releases eIF4E. eIF4E binds to eIF4A and eIF4G to form the eIF4F complex. The eIF4F complex is essential for mRNA binding and recruiting ribosomes. **b**. Step 2: The 43S preinitiation complex is formed by the assembly of the 40S ribosomal subunit with the initiation factors eIF1, eIF1A, eIF3, and eIF5. eIF2 bound to Met tRNA is subsequently recruited, which helps the tRNA in start codon recognition. This 43S complex is active but lacks mRNA for translation. **c**. Step 3: The eIF4F complex interacts with PABP on the 3’ end of the mRNA, resulting in circularized mRNA. The 43S preinitiation complex is attached to mRNA, aided by initiation factors such as eIF4G and eIF3. This step helps the attachment of the ribosome to the cap of the mRNA. eIF4A helps in unwinding mRNA, which helps the ribosome recognize the AUG codon. Met-tRNA binds with AUG, thus completing protein translation

Translation initiation requires ribosomes, which are usually reused or recycled after each cycle of protein translation. After the termination step, the post-termination ribosomal complexes (80S) are released but usually remain attached to mRNA, tRNA, and eukaryotic release factor 1 (eRF1). These complexes are recycled with the help of eIFs such as eukaryotic translation initiation factor 3 (eIF3), eukaryotic translation initiation factor 3 J (eIF3J), eukaryotic translation initiation factor 1 (eIF1), and eukaryotic translation initiation factor 1A (eIF1A) **(**Fig. 3a**).**

The eukaryotic translation initiation factor 2 (eIF2)-GTP-Met-tRNA attaches to the 40S subunit and is bound to eIF1, eIF1A, and eIF3 initiation factors, forming the 43S preinitiation complex. The exact binding position of eIF2-GTP-Met-tRNA on the 40S subunit is not known. The Met-tRNA gets superficially attached to the P-site of the ribosome complex with codon-anticodon pairing [16, 17]. While 4E-BP1 is not directly involved in the recycling of ribosomal complexes or the formation of the 43S preinitiation complex, its regulation of eIF4E controls mRNA availability for ribosome attachment and initiation (Fig. 3b).

#### Attachment of the 43S Complex to mRNA

The 43S complex binds to the 5’-UTR of mRNA, which is aided by the eIF4F, eukaryotic translation initiation factor 4B (eIF4B), and eukaryotic translation initiation factor 4H (eIF4H) in unwinding the 5’cap for ribosomal binding. The eIF4F consists of eIF4E (cap-binding protein), eIF4A (RNA helicase), and eIF4G (binds to eIF4E, eIF4A, PABP, and eIF3) **(**Fig. 3c**)**. eIF4E has two conserved tryptophan (Trp) residues on its concave side, which hold the cap of the mRNA. The nucleotides around the cap also help stabilize the binding between mRNA and eIF4E. Here, eIF4A helicase activity is increased by eIF4B, eIF4G, and eIF4H, and the affinity of eIF4E for the mRNA cap is increased by the binding of eIF4G to eIF4E. eIF4H binds at the back of eIF4A on the mRNA, which stops the mRNA from reannealing. Owing to the bulky eIF4F-eIF4B complex, the eIF4A is only bound to the 5’ end of the mRNA by the interaction of eIF4E with the cap. This step ensures that the mRNA is prepared for the attachment of the ribosome, which is aided by eIF4G and eIF3 [18, 19].

#### 5’- to 3’-mRNA Scanning

The 43S complex progresses along the mRNA until it identifies the AUG. This complex moves along the 5’-UTR, unwinding any possible secondary structures while allowing easy movement of the ribosome. Some studies have shown that the 43S complex can attach to unstructured 5’-UTR sequences and move along mRNAs without any aid from helicases or other supporting proteins. The absence of eIF1 and eIF1A nearly abolishes movement of the complex along the mRNA, whereas eIF4A, eIF4G, and eIF4E are required for its progression. It is not possible to distinguish the different functions of each initiation factor in the 43S complex, as they overlap with each other, such as attachment of the 43S complex to mRNA, ribosome recruitment, and movement along the mRNA [20, 21].

#### Initiation Codon Recognition

eIF1 helps the 43S complex identify AUG from other codons or AUG codons that are located < 8 nucleotides from the 5’ end. The complex normally recognizes the AUG start codon at + 1 to + 3 and adjacent codons such as purines at −3, and a G at + 4 can also aid in this recognition. eIF1A, closely associated with the 40S subunit, assists in stabilizing codon–anticodon binding, whereas the displacement of eIF1 produces a conformational change that locks the mRNA in position. The nucleotides at −3 and + 4 of AUG help in the selection of the initiation codon by neutralizing any conformational change during codon‒anticodon pairing with its interaction with eIF2 [22].

#### Commitment of Ribosomes to a Start Codon

After recognition of the initiation codon, eIF2 present in the eIF2-GTP-Met-tRNA 40S ribosome complex binds to eukaryotic translation initiation factor 5 (eIF5), thereby activating the GTPase activity of the γ subunit. The hydrolysis of activated eIF2-GTP, along with eIF1, helps maintain the specificity of the initiation codon and helps in the codon-anticodon pairing [16, 17, 23].

#### Ribosome Subunit Joining and Initiation Factor Displacement

Eukaryotic translation initiation factor 5B (eIF5B) plays a major role in joining the 60S subunit to the 40S complex and promoting dissociation of initiation factors such as eIF1, eIF1A, eIF2-GDP, and eIF3. While eIF5B can partially remove eIF2 from the 40S subunit on its own, complete removal occurs only in the presence of the 60S subunit. Hydrolysis of eIF5B-GTP subsequently facilitates its release from the 80S ribosome. eIF1A usually stays attached to the ribosome and dissociates later along with eIF5B. Only the dissociation of eIF3 and eIF4G is delayed, but all the other proteins attached to the 40S ribosome are removed before or during ribosome binding [16, 17, 24]. These events mark the end of translation initiation, which is followed by elongation and termination. In this review, we focus on the regulation of protein translation at the initiation stage, specifically at the eIF4E level. Hence, we have only discussed the initiation step in detail.

### Regulation of the Initiation of Protein Translation

The regulation of translation initiation is usually in two ways. One is by targeting different eIFs and regulating the whole process thereafter. The other is by the direct targeting of mRNAs by RNA-binding proteins, or microRNAs (miRNAs), which are specific to a particular mRNA. In the regulation of different initiation factors, eIF2 and eIF4F are regulated by phosphorylation, and eIF4G is regulated by irreversible proteolysis. eIF4F is usually regulated through eIF4E binding proteins, which are 4E-BP1, 4E-BP2, and 4E-BP3 [25] **(**Table 1**)**. 4E-BPs bind to eIF4E, making it unavailable to bind to eIF4G to form the initiation complex. eIF4G has a TyrXXXXLeuɸ sequence, which is highly conserved and facilitates its binding to eIF4E. The same conserved sequence is also used by 4E-BPs while binding to eIF4E, which inhibits its binding with eIF4G. Both eIF4G and 4E-BP, when bound to the conserved sequence of eIF4E, undergo structural changes involving α-helical motifs located on the dorsal region near the cap-binding site [26]. In addition to the conserved sequence, additional surfaces present on the carboxy terminus of the C motif play a role in this binding. 4E-BP-mediated inhibition of translation can be regulated by phosphorylation. Unphosphorylated 4E-BP is capable of binding to eIF4E, thus inhibiting translation, whereas the hyperphosphorylated form is incapable of binding to eIF4E [27, 28]. Many factors affect the conversion of 4E-BP to its hyperphosphorylated form, such as growth factors, cytokines, hormones, nutrient availability, and a few G protein-coupled receptor agonists and mTOR inhibitors, such as rapamycin [29, 30].

**Table 1:** A brief comparison of the structural and functional roles of the 4E-BP1, 4E-BP2, and 4E-BP3 proteins

Features	4E-BP1	4E-BP2	4E-BP3	References
Gene	EIF4E-BP1	EIF4E-BP2	EIF4E-BP3	[5, 8, 30, 31]
Protein	eIF4E-BP1	eIF4E-BP2	eIF4E-BP3	
Size	12,580 Da	12,939 Da	10,873 Da	
Amino acids	118 amino acids	120 amino acids	100 amino acids	
Majorly expressed tissue	Muscle, liver and lung	Brain	Muscle	[32, 33]
Nervous tissue expression	Moderate	High	Low	
Regulation	Controlled by mTORC1	Controlled by mTORC1	Controlled by mTORC1 and TFE3 upon extended inhibition of mTORC1	[34, 35]
Key Phosphorylation sites	Thr37, Thr46, Ser65, Thr70	Thr37, Thr46, Ser65, Thr70	Thr23, Thr32, Ser51, and Thr56 (Many are not functionally characterized yet)	[1, 29, 36, 37]
Function	Phosphorylation releases eIF4E to initiate translation. This is essential in stress conditions to regulate neuronal plasticity	Phosphorylation releases eIF4E to initiate translation. This is also essential for neuronal plasticity and is extensively linked to ischemic injury	Compensatory repressor during chronic mTORC1 inhibition	[1, 35, 38]

4E-BP1, 4E-BP2, and 4E-BP3 have similar size and amino acid sequences. 4E-BP1 and 4E-BP3 are mainly expressed in muscle, whereas 4E-BP2 is abundantly present in the brain. All three 4E-BPs are regulated by mTOR, but only 4E-BP3 is additionally regulated by TFE3 upon extended inhibition of mTOR. The phosphorylation sites in 4E-BP1 and 4E-BP2 are widely studied, whereas 4E-BP3 largely remains unexplored. All three 4E-BPs perform a single function, which is to regulate eIF4E in turn, tightly controlling cellular protein translation.

### Regulation of Protein Translation by 4E-BP1

For the first time, Blackshear et al. identified a heat-stable eIF4E binding protein in rat adipocytes. This protein was observed to be phosphorylated at specific serine and threonine residues in the presence of insulin [39]. This protein was subsequently referred to as phosphorylated heat- and acid-stable protein regulated by insulin (PHAS-1) and renamed 4E-BP1. PHAS-1 was isolated and purified from the adipocytes of male Sprague‒Dawley rats. The human 4E-BP1 gene, located on chromosome 8, spans 827 base pairs and encodes a 118-amino acid protein. Phosphorylation of 4E-BP1 affects its affinity for eIF4E [40–42]. Various stimuli, including growth factors such as insulin-like growth factor 1 (IGF1), cytokines, mitogens, G protein-coupled receptor agonists, and hormones, can induce its phosphorylation [43, 44]. This protein is selectively expressed in muscle and adipocytes.

Its capacity to be phosphorylated by growth factors but not by cAMP separates it from other known heat-stable proteins. To support this novel finding, no sequence similarity was identified with any previously characterized proteins [45]. PHAS-1 is phosphorylated by MAPK at serine 64, and different types of growth factors, such as epidermal growth factors and platelet-derived growth factors, can stimulate its phosphorylation [46]. Two groups worked on a library of proteins that can bind to eIF4E and identified 4E-BP1 and 4E-BP2. They reported that insulin induces the phosphorylation of 4E-BP1, causing its dissociation from eIF4E. This release allows eIF4E to bind mRNA, thereby promoting protein translation. Therefore, at this point, it became clear that human 4E-BP1, also known as PHAS-1, is the same protein [47, 48]

4E-BPs and eIF4G bind to eIF4E at similar binding sites [49, 50]. 4E-BPs inhibit the interaction of eIF4E with eIF4G, in turn suppressing the formation of the eIF4F complex. They share a similar conserved eIF4E-targeting sequence, Tyr-X-X-X-X-Leu-ɸ. Any change to this sequence, a deletion, or a mutation in amino acids such as tyrosine and leucine can dislodge the binding between eIF4E and 4E-BPs [26]. The eIF4E binding site attached to a synthetic peptide can also reduce the translation of the fusion protein in* in vitro* model systems [31, 51]. 4E-BP1 inhibits protein translation both in cell-free and *in vitro* model systems. The 35–85 residues that constitute the eIF4E binding site of 4E-BP1 are 100% conserved among all 4E-BPs [1, 31, 52]. NMR and circular dichroism analyses of 4E-BP1 revealed that it exists as an unstructured molecule in solution but becomes slightly ordered in the presence of mouse or yeast eIF4E [47, 52]. It binds to the convex surface that is present on the dorsal side of the cap-binding domain of eIF4E. 4E-BP1 folds into an L-shaped α-helix when it binds to eIF4E [53–56]. The eIF4E targeting sequence Tyr-X-X-X-X-Leu-ɸ in the 4E-BPs is numbered starting from Tyr (54 in 4E-BP1/2 and 40 in 4E-BP3) as 0 and gradually corresponds to ɸ as 6. X from 1 to 4 are Ala (55 in 4E-BP1/2 and 41 in 4E-BP3), Arg (56 in 4E-BP1/2 and 42 in 4E-BP3), Lys (57 in 4E-BP1/2 and 43 in 4E-BP3), and Phe (58 in 4E-BP1/2 and 44 in 4E-BP3), whereas Leu at 5 (59 in 4E-BP1/2 and 45 in 4E-BP3) and ɸ are met (60 in 4E-BP1) in 4E-BP1 and Leu (46 in 4E-BP2/3) in 4E-BP2/3 [26].

The specific residues in murine eIF4E that interact with 4E-BP1 are His37, Val69, Trp73, Leu131, Leu135, Glu132, Ile138, Glu140, and Asp147. In the yeast eIF4E ortholog, residues such as 32–50 and 62–79 are highly affected in the presence of 4E-BP2, indicating their involvement in binding to it [52, 57]. Thus, many overlapping amino acids in eIF4E are clearly involved in binding to different 4E-BPs [58, 59]. One study demonstrated that a single substitution, Trp73Ala, can inhibit the binding of 4E-BP1 and eIF4E in mice. mTOR phosphorylates the Thr37 and Thr46 sites in 4E-BP1. These amino acids are present at the N-terminus relative to the eIF4E-binding site and become phosphorylated upon serum deprivation in cell lines. The phosphorylation of Thr37 and Thr46 is important for the subsequent phosphorylation of all other sites, such as Ser65, Thr70, and Ser83, all of which are located C-terminal to the eIF4E-binding site [19, 26, 60]. Luteolin, a flavonoid, has been shown to reduce phosphorylation of 4E-BP1 at Thr 37/46 position. It is shown to provide neuroprotective effects against different neurodegenerative disorders by regulating different pathways such as mTOR, AMPK, and MAPK/ ERK. Further studies are required to validate the results through clinical trials [61, 62]. Another flavonoid, Baicalein, is known to reduce the phosphorylation of 4E-BP1 at Thr37 and Ser65. It reduces neuroinflammation and provides neuroprotection against different neurodegenerative disorders by targeting mTOR, Akt, Nuclear factor kappa B (NFκB), and Nuclear factor Erythroid 2-related Factor 2 (Nrf2) pathways. There is a need for further studies to understand the bioavailability of this compound and to unveil its molecular mechanisms to understand its full potential [63–65].

*Drosophila* 4E-BP (d4E-BP) has ~ 34% amino acid similarity with human 4E-BP1. d4E-BP was identified by its interaction with *Drosophila* eIF4E and contains a YXXXXMK domain, which differs somewhat from the equivalent mammalian domain and interacts less intensely with eIF4E compared to mammalian 4E-BP1. An increased affinity of 4E-BP for eIF4E is observed when + 5 Met and + 6 Lys are mutated to leucine in *Drosophila*. In d4E-BP, Thr37, Thr46, Ser65, and Thr70 are identical to those in human 4E-BP1, but Ser83 is a Thr residue, Ser101 is a Gln, and the Ser112 residue is absent [66, 67]. These differences may contribute to the reduced binding affinity of d4E-BP for eIF4E and highlight the evolutionary divergence of 4E-BP-eIF4E interactions between species. The yeast eIF4E translational repressor is known as Caf20. Apart from a functional eIF4E binding region, it shares no similarity with mammalian 4E-BP1. Caf20 also competes with eIF4G for binding with eIF4E, which in turn affects cap-dependent translation. Caf20 overexpression resulted in slow growth, whereas its disruption resulted in an increased growth rate in yeast [68, 69]. Another study revealed that the deletion of Caf20 did not affect the growth pattern, but it compensated for the growth pattern caused by the deletion of eIF4B and eIF4G [70]. Adenovirus infection results in hyperphosphorylation, whereas infection by poliovirus or encephalomyocarditis virus results in dephosphorylation of 4E-BP1, although the levels of protein translation were not analyzed [71–73].

Heat shock reduces the phosphorylation of 4E-BP1 in the Chinese hamster ovarian cell line and myocytes from adult rats. However, in rat hepatoma cells, heat shock leads to an increase in 4E-BP1 phosphorylation [74, 75]. Taken together, these findings highlight the complex and evolutionarily conserved mechanisms by which 4E-BP and related proteins regulate cap-dependent translation through their interaction with eIF4E and demonstrate how post-translational modifications and sequence variation can fine-tune these interactions across species and cellular conditions.

### Regulation of Protein Translation by 4E-BP2

Like 4E-BP1, 4E-BP2 was also shown to inhibit cap-dependent translation [47]. These proteins were shown to share a similar binding motif with eIF4G [51]. 4E-BP2 is ubiquitously expressed in our bodies, and it is the most widely expressed 4E-BP homolog in the brain. The 4E-BP2 gene consists of just 3 exons but spans ~ 20 kb on chromosome 10. It is expressed as a 7491 bp mRNA transcript and encodes a 120 amino acid protein [32]. 4E-BP2 is also stimulated and inhibited by the same molecules and conditions as 4E-BP1. There are fewer phosphorylation sites in 4E-BP2 than in 4E-BP1, as determined by tryptic mapping and isoelectric focusing [76].

When fluorescence titration was employed to analyze the binding of 4E-BPs, 4E-BP2 showed a greater preference for binding to eIF4E than did 4E-BP1 or 4E-BP3. In contrast, the same study revealed no significant difference in the binding preferences of different 4E-BPs toward eIF4E when surface plasmon resonance was used. These contradictory differences between the two methods may be due to differences in the time points of observation or due to analysis of local or overall interactions of 4E-BPs. Molecular dynamics (MD) simulation studies have shown that the mRNA cap helps stabilize the 4E-BP-eIF4E complex and that 4E-BP binding to eIF4E helps stabilize the cap binding pocket of eIF4E [25]. The preferential binding of eIF4E to 4E-BP2 over 4E-BP1 can be explained by differences in their amino acid sequences at positions 60–63. 4E-BP1 consists of Met-Glu-Cys-Arg, whereas 4E-BP2 has Leu-Asp-Arg-Arg. The arginines in 4E-BP2 form a more stable hydrophobic interaction around the Trp73 indole ring, making it more preferred over 4E-BP1. 4E-BP2 contains a highly variable secondary structure, and its interaction with eIF4E depends not only on the Tyr-X-X-X-X-Leu-ɸ residue but also on highly conserved IPGVT sequences [30, 77].

Rapamycin inhibits the phosphorylation of 4E-BPs through mTORC1 activity [78–80]. However, 4E-BP1 phosphorylated at Thr37 and Thr46 can resist inhibition by rapamycin, resulting in sustained growth and proliferation despite rapamycin treatment. However, the phosphorylation of 4E-BP2 at Thr37 and Thr46 is strongly inhibited by rapamycin in 4E-BP2-abundant cell types such as lymphocytes [81]. Phosphorylation of 4E-BP2 at Thr37 and Thr46 decreases its affinity for eIF4E, modulating the interaction. However, mutant forms such as Y54A and L59A are prone to misfolding. The phosphorylation of 4E-BP2 at Thr37 and Thr46 introduces negative charges to 4E-BP2, which weakens its interaction with eIF4E by promoting electrostatic repulsion and altering the eIF4E-binding site within its binding pocket [82–84]. The stabilization of the folded 4E-BP2 protein is facilitated by phosphorylation at Thr37 and Thr46. These phosphorylation sites increase protein folding stability [36, 85]. When 4E-BP2 is phosphorylated at these two sites, it undergoes a conformational change into a β-sheet, making it more flexible for binding with other proteins. This β-sheet structure is formed by Pro18-Arg62. Hydrogen bonding occurs between pThr 46 and Gly 48, between Thr 50, and between pThr 37 and Gly 39; Thr 41 plays a major role in the initiation of the β-sheet turn. There are four β strands in 4E-BP2, in which β1 and β4 are formed early, followed by β2 and β3 [36, 85–88].

4E-BP2 knockout (KO) results in memory and spatial learning defects in mice due to alterations in protein translation in the hippocampal region of the brain [38]. These 4E-BP2 constitutive KO mice presented a reduction in motor skills, as analyzed by the rotating rod experiment. Anxiety levels were normal according to the elevated plus maze and light‒dark chamber exploration tests, whereas the chamber-based exploratory task revealed that 4E-BP2 KO mice preferred to explore new areas and objects. The conditioned taste aversion test revealed that the memory of the 4E-BP2 KO mice was heightened, indicating that the amygdala region of the brain, rather than the hippocampus, was unaffected. The mice showed no change in performance in the passive avoidance test, suggesting that the capacity to learn is intact in 4E-BP2 KO model mice [89]. Both the 4E-BP1 and 4E-BP2 KO mice presented impaired granulomonocytic differentiation, indicating the importance of translation and translation factors in hematopoietic differentiation [90]. 4E-BP2 KO mice also presented increased synaptic function and long-term plasticity [91]. 4E-BP1 and 4E-BP2 play their respective independent roles in sleep‒wake cycle regulation in humans, as confirmed in 4E-BP1 and 4E-BP2 KO mice [92]. Conditional KO of 4E-BP2 in Purkinje neurons resulted in increased action potential with normal social interaction but reduced spatial memory in mice. The absence of social deficit suggests that autistic behavior in these mice is not regulated by the mTORC1-4E-BP2 axis [93]. 4E-BP2 deletion in excitatory neurons and astrocytes did not result in autistic-like behavior in mice, whereas deletion in inhibitory neurons resulted in autistic-like behavior, such as a decrease in vocalization and social interaction. These results indicate that global 4E-BP2 KO effects arise from multiple cell types rather than a single population [94].

Deamidation of 4E-BP2 occurs spontaneously and may contribute to the regulation of 4E-BP2 in the brain, where it converts asparagine to aspartate or isoaspartate [95, 96]. This reduces the interaction of 4E-BP2 with eIF4E, which in turn affects synaptic transmission in excitatory neurons [97]. The protein L-isoaspartyl methyltransferase (PIMT) helps in the clearance of iso-aspartates formed by deamidated 4E-BP2 in the brain. 4E-BP2 may serve as a substrate for PIMT, which converts iso-aspartate residues back to aspartate, thereby influencing synaptic transport and postnatal brain development [98]. The N99 and N102 residues of 4E-BP2 are deamidated, especially in neuron regulation of protein translation in the postnatal brain [99].

Ischemia in the brain leads to neuronal loss or death, further leading to the inhibition of protein translation in affected areas. As 4E-BP2 is the dominant member of the 4E-BP family expressed in the brain, understanding its interactome might help in the identification of biomarkers or therapeutic targets in neuronal stress and ischemia. The phosphorylation of 4E-BP2 does not significantly alter its binding to eIF4E during stress conditions. Compared with that in control tissues, the 4E-BP2 interactome in brain tissues from patients with ischemia varied considerably. Proteins such as enolase-1, Rho guanine nucleotide dissociation inhibitor (Rho-GDI), Heat shock cognate protein 70 (HSC70), Nucleoside diphosphate kinase A (NDKA), Dystrophin-related protein 2 (DRP2), Ubiquitin C-terminal hydrolase L1 (UCHL1), and Adenylate kinase 1 (ADK1) were expressed at higher levels in resistant brain tissues than in ischemic regions. Moreover, at the levels of superoxide dismutase 1 (SOD1) and DRP2 increased with ischemia‒reperfusion. HSC70 is a molecular chaperone essential for protein folding, and UCHL1 is involved in protein degradation and optimal synapse function. The differential expression of these proteins, along with that of 4E-BP2, explains their involvement in proteostasis and synaptic maintenance. Rho-GDI and DRP2 are involved in cytoskeletal maintenance, suggesting that 4E-BP1 could also influence neuronal structure and morphology. These interactions may indicate that impaired cytoskeletal structures lead to neuronal death. SOD1 protects neurons from oxidative stress, which may be exacerbated by ischemia. Its interaction with 4E-BP2 may suggest oxidative damage in specific regions of the brain. These proteins can be further studied as biomarkers and may play a significant role in the regulation of protein translation, especially under stress conditions such as ischemia [99–102].

Upon further study, DRP2 was found to interact with both 4E-BP2 and eIF4E and play important roles in neuronal survival during cerebral ischemia. The hypothesized mechanism involves the interaction of hypophosphorylated DRP2 with 4E-BP2 during cerebral stress. This interaction increases protein translation, thereby supporting neuronal survival. During ischemic reperfusion, hyperphosphorylated DRP2 becomes inactive, allowing 4E-BP2 to inhibit protein translation and leading to neuronal death. Although these findings are intriguing, the exact mechanism remains unclear [103].

In neuronal stem cell differentiation, 4E-BP2 plays a major role, striking a balance between NSC differentiation and the production of neurons. The activation of 4E-BP2 results in outcomes similar to those of decreased mTORC1 by suppressing translation, leading to the inhibition of NSC differentiation and reduced neuronal production. However, 4E-BP2 knockdown resulted in increased mTORC1 activity via the promotion of NSC differentiation and the production of neurons [104].

### Regulation of Protein Translation by 4E-BP3

A previously unknown ~ 10,873 Da protein, similar to the other 4E-BPs, was identified and named 4E-BP3. The gene encoding 4E-BP3 is composed of three exons and is located on chromosome 5 in humans. Its transcript is ~ 693 bp long and encodes a 100 amino acid protein. The first intron is conserved in *Drosophila*, mice, and humans and across the other 4E-BPs, suggesting that key regulatory elements may be present in this region. The phosphorylation sites and regulation of 4E-BP3 have not been studied in detail. Despite its minimal expression in the brain, 4E-BP3 remains active and performs protein translation when 4E-BP1 and 4E-BP2 are inactive during prolonged mTORC1 inhibition. The transcription factor E3 (TFE3) binds to the promoter region of the 4E-BP3 gene and increases its transcription, which is essential for this compensatory role of 4E-BP3. This regulation maintains tight control over protein translation during sustained mTORC1 inhibition [34, 35].

Like its homologs, 4E-BP3 can reduce protein translation levels by binding to eIF4E. Its highest protein expression is found in skeletal muscle and the heart, whereas the brain has the lowest expression level. Mutations such as Y40A and L45A were unable to bind to eIF4E. At the subcellular level, this protein is found in both the cytoplasm and the nucleus [31, 37, 105]. The binding of 4E-BP3 to eIF4E is influenced by nutrient availability. The N-terminus of this protein differs from those of other human homologs. Like 4E-BP1 and 4E-BP2, 4E-BP3 cannot readily dissociate from eIF4E in the presence of insulin, explaining the importance of a full N-terminal region. This may explain why 4E-BP3 regulates protein translation under different nutrient-limiting conditions. The N-terminal residues of eIF4E also play an important role in its preferential binding to 4E-BP2 over 4E-BP1. These residues do not play a role in binding to 4E-BP3, as the deletion of N-terminal residues in eIF4E did not affect binding to 4E-BP3 [106, 107].

4E-BP3 affects the nuclear‒cytoplasmic transport of mRNAs, including those involved in protein translation, such as cyclin D1 [108, 109]. This does not affect the cellular mRNA levels of cyclin D1 but specifically impacts its transport to the cytoplasm [109]. In zebrafish, 4E-BP3 helps maintain muscle fiber size by regulating protein synthesis. It regulates the translation of Myocyte enhancer factor 2C (Mef2c), an essential transcription factor for muscle development [33]. When mTORC1 activity is inhibited, 4E-BP1 and 4E-BP2 are dephosphorylated and bind to eIF4E, thereby reducing protein translation. However, upon sustained mTORC1 inhibition, 4E-BP1 and 4E-BP2 expression or activity diminishes, and 4E-BP3 is upregulated, subsequently taking over its role in repressing translation. The transcription factor TFE3 helps increase 4E-BP3 levels, which in turn help regulate the translation of essential proteins such as cyclins. In 4E-BP3 KO, increased translation and resistance to long-term exposure to mTOR inhibitors were observed [34, 35].

**Fig. 4:**
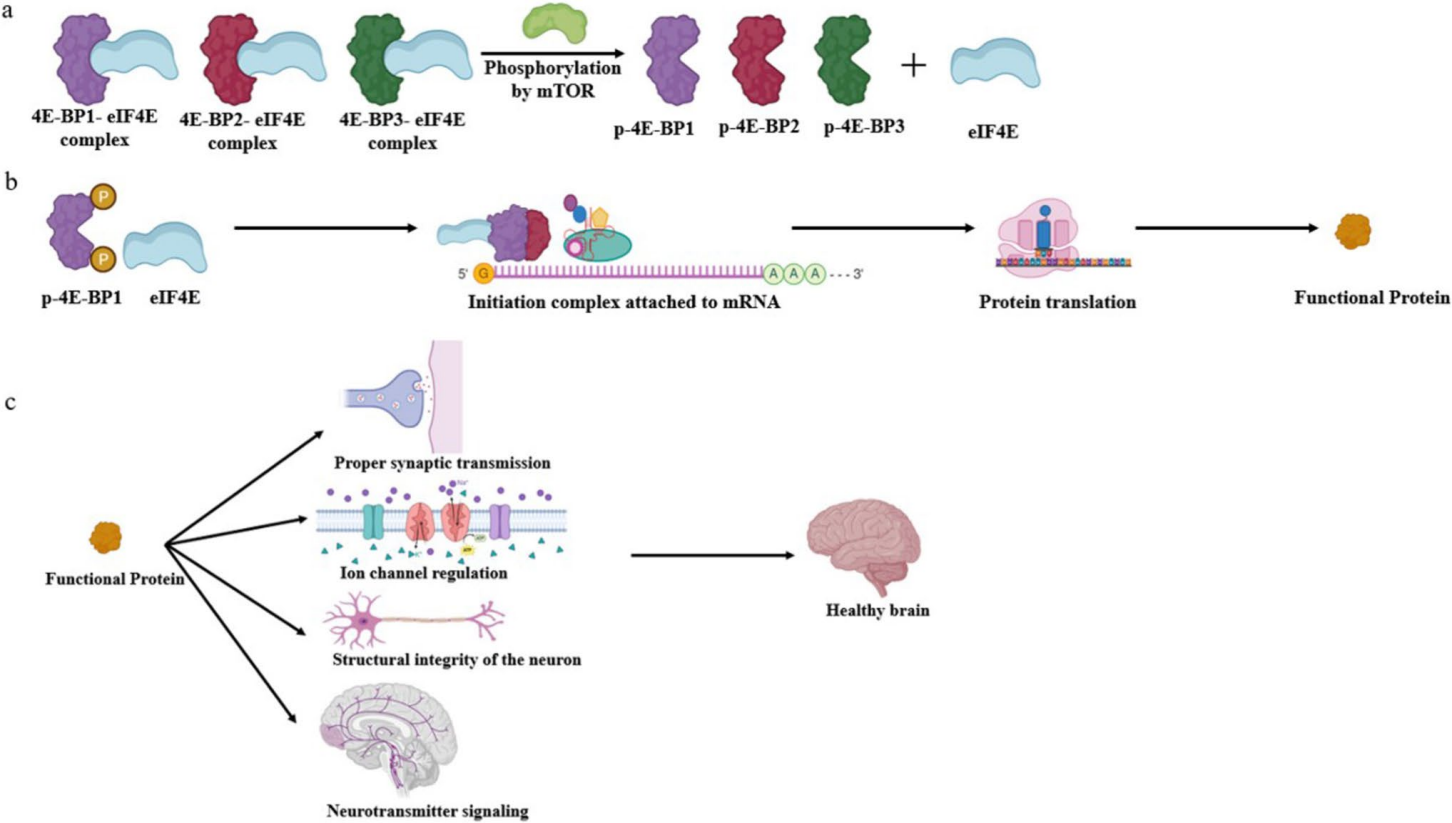
Endogenous mTOR signaling pathway and its effects on neuronal health. **a**. The mTOR signaling pathway facilitates protein synthesis, as it phosphorylates 4E-BPs, leading to their detachment from the eIF4E-4E-BP complex. **b**. The separated eIF4E joins the initiation complex, which attaches to the mRNA cap structure, allowing the translation of the mRNA into a functional protein. This tightly regulated mTOR activity is essential for neurogenesis, dendritic growth and maintenance, synaptic plasticity, neurotransmitter regulation, etc. **c**. The mTOR pathway regulates proper synaptic transmission, which helps neurons communicate effectively through neurotransmitter release. Proteins also control inhibitory and excitatory signals through ion channels. Structural proteins are essential for maintaining the integrity of neurons and dendrites. Overall, tightly regulated protein translation by mTOR helps in maintaining neuronal homeostasis, protecting brain health, and prevention from neurological disorders

The critical regulation of protein synthesis is essential for cell homeostasis, especially in the brain, where tightly regulated protein translation is necessary to maintain several neuronal functions for optimal functioning (Fig. 4). Dysregulation can contribute to a range of neurological disorders. Many neurodegenerative diseases are characterized by specific hallmark protein aggregations due to the production of misfolded proteins. Protein synthesis is indirectly connected to autophagy, helping in the clearance of misfolded proteins, but dysregulation can lead to aggregation. Deregulated protein synthesis in synapses can cause changes in memory and synaptic plasticity, which are observed in several neurodevelopmental disorders. Studying protein translation related to the maintenance of brain function and the development of disorders related to this process will help researchers understand the complex molecular mechanisms involved and identify potential drug targets. In this review, we focus on a downstream target of mTOR, 4E-BPs, which are a negative regulator of protein translation, and their regulation in different disorders.

### Physiological Stressors and 4E-BP Signaling

In this section, we discuss a few important physiological stressors and their regulatory effects on 4E-BPs.

#### Oxidative Stress

Oxidative stress is caused by dysregulation of the mechanism of reactive oxygen species (ROS). Increased ROS and decreased ATP levels activate AMPK. AMPK plays an important role in the ROS mechanism and acts as a checkpoint [110, 111]. It inhibits the mTORC1 complex by phosphorylating its TSC2 and RAPTOR subunits. This leads to the dephosphorylation of 4E-BPs, which inhibit protein translation, as explained in Sects. "Regulation of protein translation by 4E-BP1", "Regulation of protein translation by 4E-BP2", and "Regulation of protein translation by 4E-BP3". The inhibition of global translation saves energy and reduces the burden of stress on the cell. Now the cell can induce the translation of mRNAs, which are essential for stress response, antioxidant enzymes, and a few essential proteins, through alternative mechanisms. In neurodegenerative disorders such as Alzheimer's disease (AD) and Parkinson's disease (PD), protein accumulation activates this pathway through AMPK. This is a mechanism by which cells provide neuroprotection against ROS [112–114].

#### Circadian Rhythms

Circadian rhythms are 24-hour repetitive cycles built into our bodies. There are proteins that regulate all major physiological processes and synchronize them to this cycle [115]. These include essential proteins such as circadian locomotor output cycles protein kaput (CLOCK) and brain and muscle ARNT-like 1 (BMAL1). Dysregulation of these proteins is linked to a decreased life span [116]. These proteins function through the mTOR-4E-BP axis to regulate global translation. mTOR regulates circadian rhythms in the suprachiasmatic nucleus (SCN) of the brain [117]. The translation of essential proteins such as vasoactive intestinal peptide (VIP) is regulated by the phosphorylation of 4E-BP1 in the SCN. Deletion of 4E-BP1 is known to affect the circadian rhythm of the SCN. BMAL1 is a negative regulator of mTORC1 and, in turn, controls 4E-BP1. Loss of these genes, especially in neurons, leads to synapse loss and causes neurodegeneration. Circadian rhythm alterations are known to accelerate protein accumulation and neuroinflammation [118–120].

#### Chronic Stress

Long-term experience of physical or mental trauma due to exposure to stressors can lead to chronic stress. Glucocorticoids are released in response to chronic stress through the hypothalamic pituitary adrenal axis in the brain. Cortisol is a glucocorticoid hormone that is commonly known as a stress hormone that activates the fight or flight response [121]. Sustained exposure to cortisol keeps 4E-BP1 in its inactive state by regulating mTORC1. This causes aberrant activation of protein translation, leading to neuronal overactivation [122]. Neuronal hyperexcitability is related to many neurological conditions and psychiatric disorders. Further studies are needed to elucidate the intricate mechanisms involving chronic stress and 4E-BP1 and to determine whether the mTOR-4E-BP1 pathway can be used as a therapeutic target [123, 124].

## Neurodegenerative Disorders and 4E-BP’s

### Alzheimer's Disease

Alzheimer's disease (AD) is a neurodegenerative disorder that is characterized by amyloid-beta plaques and tau protein aggregation, causing neuronal damage in areas such as the hippocampus [125, 126]. It is also characterized by cognitive deficits and specific behavioral and psychiatric symptoms [127]. The primary risk factor for AD is age, but other minor risk factors include complex genetics, lifestyle, and environmental factors such as alcohol abuse, physical inactivity, diabetes, and hypertension. Females are affected more often than males [127]. Only 1–5% of AD cases are familial (FAD), and Mendelian mutations in just three genes, Amyloid protein precursor (APP), presenilin-1 (PSEN1), and presenilin-2 (PSEN2), account for most FAD cases. Cholinesterase inhibitors and *N*-methyl d-aspartate antagonists are used to alleviate AD symptoms. The hallmarks of this disease are neuroinflammation, excessive reactive oxygen species, mitochondrial dysfunction, progressive dementia, and aberrant mTOR activity [128, 129].

Aβ is known to increase the PI3K/Akt/mTOR axis in AD patients [130]. The aggregation of amyloid-beta plaques and tau protein and dysregulation of synaptic plasticity are associated with impaired protein translation. Age-related reduction in the efficiency of autophagy also contributes to protein accumulation in brain tissues [131]. Therefore, there is a need to study protein translation, with a focus on 4E-BPs in the context of AD [132, 133].

When the upstream and downstream proteins of the mTOR and Akt pathways were analyzed in AD mouse models, there was a 34% reduction in mTOR activity was detected in the cortex of APPSL/PS1 double transgenic mice. A similar trend was found in neuroblastoma cell lines upon treatment with Aβ. Upon the progression of neurofibrillary degeneration, mTOR levels were increased in the AD brain, whereas phospho-mTOR (p-mTOR) levels were unchanged except at the autophosphorylation site Ser2481, which was significantly increased [134, 135]. This increase in autophosphorylation correlated positively with tau levels in the brain. Upon the progression of neurofibrillary degeneration, 4E-BP1 levels were decreased in the cortex of the brains of AD transgenic mice, and phospho-4E-BP1 (p-4E-BP1) (Thr70 and Ser65) levels were increased. This increase in p-4E-BP1 correlated with mTOR autophosphorylation levels and tau levels in the AD brain. This causes eIF4E to activate protein translation, which explains its involvement in AD pathophysiology [136].

The cortex of an AD patient showed a significant increase in the levels of p-mTOR (Ser2481) and p-4E-BP1 (Thr70 and Ser65). It also showed increased mTOR autophosphorylation at Ser2481, whereas other phosphorylation sites, such as Ser2448 by PI3K, did not significantly change. 4E-BP1 phosphorylation in AD might be assisted by an mTOR autophosphorylation-dependent pathway [137]. In the post-mortem tissue of AD patients with amnestic mild cognitive impairment (MCI) and pre-clinical AD (PCAD), there was a significant increase in the levels of p-mTOR, p-70S6K (Thr389) and p-4E-BP1 (Thr36) in AD and MCI patients compared with controls. The increase in the PI3K/Akt/mTOR pathway in AD and MCI patients but not in PCAD patients may be due to the lack of excessive oxidative stress [138]. In a rat model of AD, increased levels of p-mTOR, p-4E-BP1, p70 ribosomal S6 protein kinase 1, Interleukin 1beta (IL-1β), Interleukin 6 (IL-6), and Tumor necrosis factor-alpha (TNF-α) were observed [139]. Similar results were observed when neuronal cells were treated with 40 Hz gamma frequency. This stimulation has been shown to reduce the secretion and aggregation of Aβ and p-tau and may influence neuronal activity [140].

All the above findings suggest an increase in the phosphorylation of mTOR and 4E-BP1 in AD mice and in patient samples, which correlate with tau protein pathophysiology, suggesting that inactivation of 4E-BP1 through phosphorylation causes eIF4E to participate in protein translation, which in turn increases the synthesis of tau protein [141]. mTOR inhibitors that decrease the phosphorylation of 4E-BP1 reduce tau synthesis and its aggregation in cells. Together, these findings indicate that the mTOR/4E-BP1 axis plays a central role in tau protein homeostasis and Alzheimer’s disease pathogenesis [142–144].

### Parkinson’s Disease

Parkinson's disease (PD) is essentially a progressive neurodegenerative disorder specific to a certain part of the central nervous system, termed the substantia nigra, which is primarily responsible for motor functions. Major symptoms include bradykinesia (slowness of movement), tremors, rigidity, and postural imbalance. Secondary symptoms such as depression, cognitive changes, sleep disturbances, and autonomic problems may also contribute to PD patients [145, 146]. The pathophysiology of PD is primarily due to the degeneration of dopaminergic neurons in the substantia nigra followed by abnormal intracellular protein aggregates, particularly alpha-synuclein, which forms Lewy bodies. Risk factors for PD include age and genetic susceptibility, and environmental agents, such as pesticide exposure or brain trauma, may also lead to PD. Treatment includes levodopa, followed by other medications to increase the activity of dopamine. Deep brain stimulation (DBS) is also used in rare cases for symptomatic relief.

**Fig. 5:**
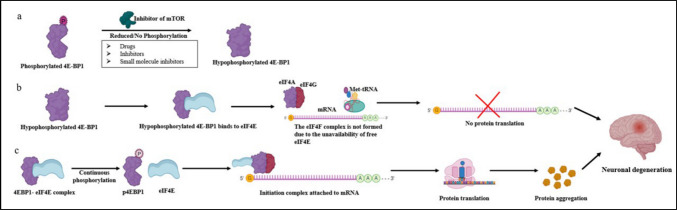
Overview of the dysregulated mTOR pathway and its effect on the human brain. The mTOR signaling pathway regulates protein synthesis through the phosphorylation of downstream targets such as 4E-BP1 in response to exogenous stimuli such as growth factors and energy signals, promoting neuronal growth, synaptic plasticity, and brain health. Dysregulation of mTOR signaling can disrupt neuronal functions and lead to neurodegenerative diseases or developmental disorders. **a**. Phosphorylation of 4E-BP1 can be inhibited by different drugs, inhibitors, and small molecules. These target 4E-BP1, which prevents its phosphorylation and, in turn, the release of eIF4E from the 4E-BP1-eIF4E complex. **b**. 4E-BP1-bound eIF4E cannot participate in the formation of the initiation complex, which in turn leads to the inhibition of protein translation. Insufficient protein synthesis causes impaired neuronal regulation and maintenance due to the lack of essential proteins. This leads to delayed synaptic transmission, compromised neuronal structural integrity, and neurotransmitter regulation. This leads to neuronal degeneration in patients. **c**. Drugs, inhibitors, and small molecules can bind to 4E-BP1 and prevent it from binding to eIF4E. This causes an aggressive increase in protein translation, which may cause overproduction of essential proteins or dysfunctional proteins, leading to aggregation. These protein aggregates cause neuronal stress and inflammation, triggering neuronal death. The accumulation of proteins is a hallmark of many neurodegenerative disorders, such as AD, and PD is characterized by neuronal loss and cognitive decline as the disease progresses

The tightly regulated mTOR pathway and protein translation balance protein synthesis and degradation, preventing the accumulation of toxic aggregates, which are essential for the healthy maintenance of neurons [147]. Dysregulation of mTOR signaling, either hyperactivation or suppression, disrupts proteostasis, impairs autophagy, and contributes to neurodegenerative diseases (Fig. 5) [148].

Familial forms of PD represent ~ 10–15% of all cases. Among the familial forms, LRRK2 gene mutations represent the most common cause, accounting for 2–40% of all familial PD cases, with the exception of rare early-onset cases [149]. 4E-BP1 is phosphorylated by LRRK2 directly at different sites, such as Thr37, Thr46, and Ser65, as observed in cell line models and *Drosophila*. Loss of dLRRK2 suppressed growth in *Drosophila,* and the overexpression of mutant d4E-BP1, which has a strong affinity for eIF4E, caused a mild reduction in eye size and a moderate reduction in wing size. When hLRRK2 was knocked down, p-4E-BP1 (Thr37/Thr46) levels were reduced. This regulation was mTOR independent, as the levels of mTOR remained constant under both conditions, suggesting a direct interaction between LRRK2 and 4E-BP1 [150].

**Fig. 6:**
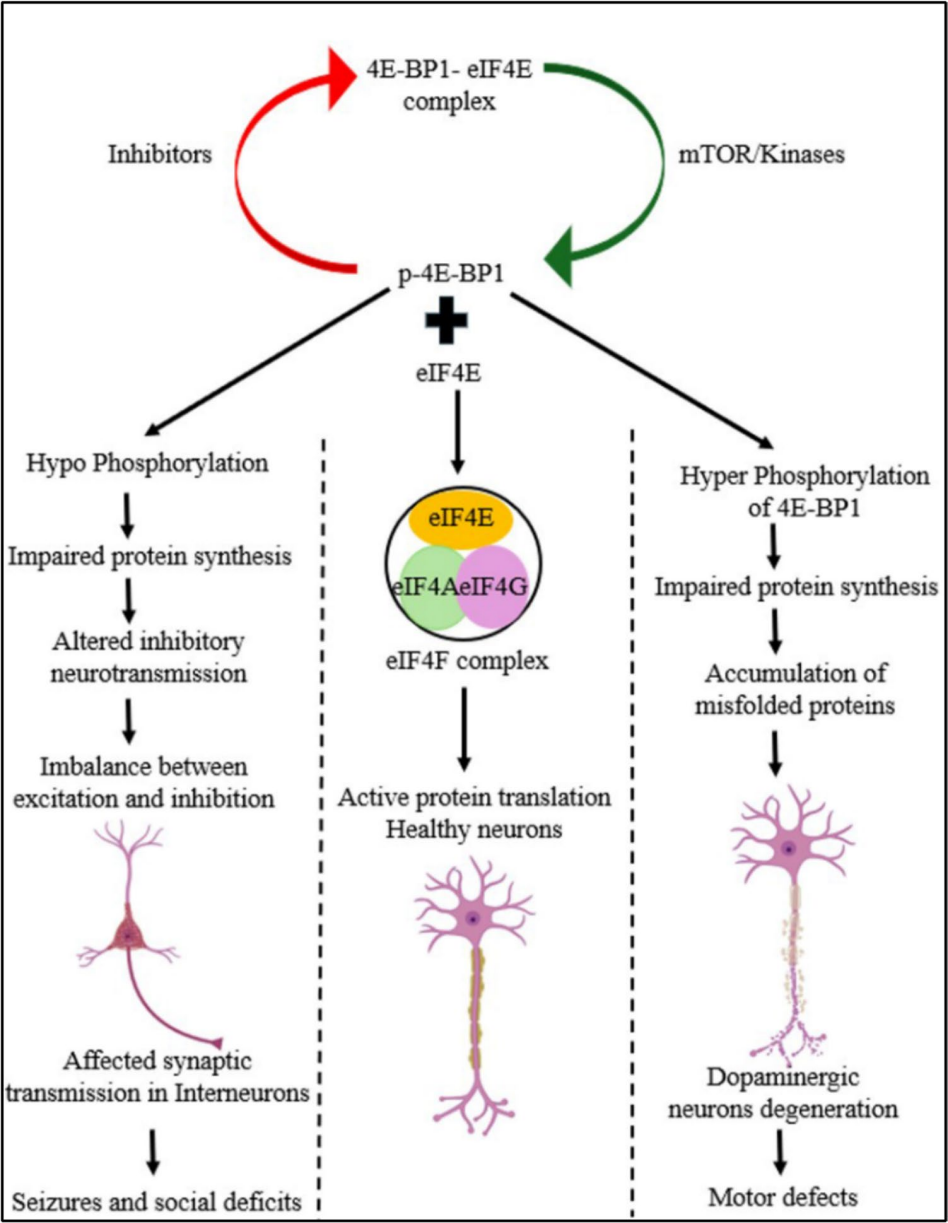
Effect of 4E-BP dysregulation in different neuronal types. Under normal conditions, tightly regulated protein translation is maintained by mTOR and its downstream target 4E-BPs. mTOR phosphorylates 4E-BP1, which triggers the release of eIF4E. Unbound eIF4E binds with eIF4A and eIF4G to form the eIF4F complex. This complex plays an essential role in the initiation of translation. This inbuilt mechanism is the basis for the healthy functioning of our nervous system, keeping all the misfolded proteins in check. Dysfunction of this system leads to either hyperphosphorylation or hypophosphorylation of 4E-BPs. Hyperphosphorylation leads to hyperactivated protein translation, leading to the aggregation of misfolded proteins, as in several neurodegenerative disorders, such as PD. This leads to degeneration of dopaminergic neurons, leading to motor deficits. Hypophosphorylation of 4E-BP1 leads to dysregulated synaptic transmission, leading to seizures and social deficits, as in autism spectrum disorders

Only a few mutants of LRRK2 target the insulin signaling pathway to phosphorylate 4E-BP1, but others do not. This finding confirms that LRRK2 deregulates protein translation by reducing the ability of 4E-BP1 to inhibit initiation, thereby contributing to neuronal dysfunction in PD [150] (Fig. 6). *Thor* is the gene that encodes the mammalian 4E-BP1 ortholog in *Drosophila;* PINK1^B9^ and Park^25^ are null mutations in the PINK1 and Parkin genes, which are often used to study the loss-of-function effects of these proteins [151, 152]. The Thor^2^ (null allele) homozygous fly mutants are viable and fertile, whereas the combination of Thor^2^ and Park^25^ or Thor^2^ and PINK1^B9^ double mutants is lethal [153]. Strikingly, 4E-BP1 overexpression partially rescues a range of neuromuscular defects, including climbing and flight defects, muscle degeneration, mitochondrial disruption, and dopaminergic neuronal loss, in parkin/Pink1 double mutant flies. In these mutants, p-4E-BP1 is reduced along with p-Akt1, indicating that the Akt/mTOR pathway is downregulated and that when rapamycin is supplemented through food, neurodegeneration is suppressed completely. They knocked down the Atg5 gene to determine whether the beneficial effects were related to autophagy and reported that only rapamycin was beneficial in double mutants, indicating that protein translation is the sole reason for this protection [152, 153]. ^33^P-scintillation counting revealed that LRRK2 autophosphorylation is 20 times more efficient than 4E-BP phosphorylation under similar conditions [154]. In HEK293 cells, neither stable LRRK2 clones nor transient overexpression of LRRK2 wild-type (WT) or pathogenic mutants (G2019S and R1441C) led to an increase in 4E-BP phosphorylation. They also reported that MAPK14/P38α can phosphorylate 4E-BP better than LRRK2 at the same site and concluded that 4E-BP phosphorylation *in vitro* is possible with p38-mediated cell stress rather than via direct LRRK2 activity and that direct interaction between 4E-BP and LRRK2 remains unclear [155]. Human brain tissues (basal ganglia and frontal cortex) from PD patients with mutations in the LRRK2 gene were analyzed, and there was no difference in the phosphorylation of 4E-BP1 between PD patients and controls. LRRK2 overexpression did not affect the phosphorylation level of 4E-BP1 in primary cortical neurons. In LRRK2 WT or KO mouse brains, the levels of 4E-BP1 and phosphorylation at T37/46 were not altered, indicating that LRRK2 is not an essential kinase that targets 4E-BP1 [156].

Under stress, PINK1 activates the HIF-1α pathway through 4E-BPs. Many studies connect PD to HIF-1α, a transcriptional factor that, under hypoxia, upregulates HRE genes, which convert glucose from oxidative metabolism to glycolytic metabolism. In addition, during hypoxia, PINK1 deficiency inactivates HIF-1α through a decrease in protein translation by hyperphosphorylating 4E-BP1. eIF4E, eIF4G and 4E-BP1 expression did not change in PINK1-KO mice. During hypoxia, PINK1^−/−^ MEFs presented increased levels of the hyperphosphorylated γ-form, whereas PINK1^+/+^ MEFs presented increased levels of the unphosphorylated α-form 4E-BP1. Compared with the PINK1^-^/^-^ MEFs, the PINK1^+/+^ MEFs presented increased levels of the hyperphosphorylated α-form of 4E-BP2. This finding explains the essential interaction between PINK1 and 4E-BP1 or 4E-BP2, suggesting that modulating this pathway could be a potential therapeutic strategy for PD [157].

p-4E-BP1 levels are dramatically increased in the substantia nigra and striatum of MPTP-induced PD mice but are drastically reduced following rapamycin treatment [158]. Six-week-old Sprague–Dawley rats were treated with 6-hydroxydopamine (6-OHDA) to observe the effect of L-DOPA on circadian rhythm via analysis of the core clock proteins and ERK1/2-mTOR proteins. The rats were treated intraperitoneally once/day for 21 days with L-DOPA, benserazide, or a D1R agonist (binds to D1 dopamine receptors). They divided the rats into control and five test groups: (i) 6-OHDA (6.25 mg/kg); (ii) 6-OHDA + L-DOPA (25 mg/kg); (iii) SKF38393 (1.5 mg/kg); (iv) DIR agonist; and (v) SCH23390 (0.25 mg/kg) (L-DOPA + benserazide + D1R agonist). They reported that total 4E-BP1 levels were constant across all groups. However, p-4E-BP1 levels were most elevated upon the addition of L-DOPA to the striatum of 6-OHDA lesioned rats, followed by SKF38393-treated rats, whereas the SCH23390 group showed a reduction in p-4E-BP1. SCH23390 can decrease the activity of p-4E-BP1 induced by L-DOPA in 6-OHDA lesioned rats. Thus, L-DOPA causes overactivation of proteins in the ERK1/2-mTOR pathway via D1R [159].

After analyzing all the information, it was hypothesized that reduced protein translation can be beneficial against PD pathology. The 4E-BP1-overexpressing mouse model showed a significant decrease in protein translation. When the primary neurons from these mice were treated with rotenone, maneb, or paraquat, there was significant protection. These neurons showed decreased formation of Lewy neurites upon treatment with α-synuclein preformed fibrils. [160]. Similar results were reported in transgenic PD A53T mice, the Drosophila PD model, and postmortem brains of PD patients [161]. These results suggest that 4E-BP1 may be a prospective drug target against PD **(**Table 2**)**.

**Table 2:** Cumulative assessment of 4E-BPs in different neurological disorders

Molecule	Mechanism of action/Key target pathways	Disease	Type of the disease	Reference
4E-BP1	4E-BP1 levels were decreased in the cortex of the AD transgenic mouse brain, and p-4E-BP1 (Thr70 and Ser65) levels were increased. This increase in p-4E-BP1 correlated with mTOR autophosphorylation levels and tau levels in the AD brain	AD	Neurodegenerative	[136, 137]
4E-BP1	Increased p-4E-BP1 at Thr36 leads to activation of the PI3K/Akt/mTOR pathway and causes oxidative stress	AD	Neurodegenerative	[138]
4E-BP1	Increased p-4E-BP1 with p-mTOR in the hippocampus of AD rat models drives neuroinflammation	AD	Neurodegenerative	[139]
4E-BP1	40 Hz Gamma stimulation in SHSY-5Y cells increased p-4E-BP1, indicating activation of the mTOR pathway, which may contribute to AD pathology	AD	Neurodegenerative	[140]
4E-BP1	4E-BP1 is phosphorylated by LRRK2 directly at different sites such as Thr37, Thr46, and Ser65 as observed in cell line models and *Drosophila*	PD	Neurodegenerative	[150]
4E-BP1	Genetic or rapamycin-induced 4E-BP1 activation restores mitochondrial function and mitigates the pathology in PINK1 and Parkin mutant models	PD	Neurodegenerative	[153, 154]
4E-BP1	Human brain tissues (basal ganglia and frontal cortex) from PD patients showed no difference in the phosphorylation of 4E-BP1 compared to controls	PD	Neurodegenerative	[156]
4E-BP1	PINK1 deficiency inactivates HIF-1α through a decrease in protein translation by hyperphosphorylating 4E-BP1	PD	Neurodegenerative	[157]
4E-BP1	Rapamycin was able to reduce p-4E-BP1 levels in the MPTP mouse model	PD	Neurodegenerative	[158]
4E-BP1	L-DOPA treatment in the 6-OHDA-induced PD model significantly increased p-4E-BP1 level via ERK1/2-mTOR activation	PD	Neurodegenerative	[159]
4E-BP1	Primary neurons from the 4E-BP1 overexpression mice model showed decreased formation of Lewy neurites upon treatment with α-synuclein preformed fibrils	PD	Neurodegenerative	[160]
4E-BP1	α-synuclein accumulation in PD is directly linked with increased 4E-BP1 phosphorylation via mTOR activation in different PD models	PD	Neurodegenerative	[161]
4E-BP1	mTOR suppression was directly linked with ASD pathology via the 4E-BP1 downregulation	Autism Spectrum Disorders	Neurodevelopmental	[168]
4E-BP1	Increased protein translation via dysregulated mTOR-eIF4E-4E-BP1 activity contributed to intractable epilepsy	Epilepsy	Neurodevelopmental	[176–178]
4E-BP1	Inhibition of the mTOR pathway prevented ketamine’s antidepressant effects in depression models. An NR2B agonist, which functions similarly to ketamine, was found to activate mTOR, 4E-BP1, and p70S6K	Depression	Neuropsychiatric	[196]
4E-BP1	LY341495, an mGluR2/3 antagonist targets the mTOR/4E-BP1 axis similar to ketamine	Depression	Neuropsychiatric	[197]
4E-BP1	Antidepressants such as escitalopram, paroxetine, and tranylcypromine induced increased levels of mTOR, 4E-BP1, and p70S6K, whereas fluoxetine, sertraline, and imipramine did not affect the mTOR pathway	Depression	Neuropsychiatric	[198]
4E-BP1	Chronic treatment with fluoxetine upregulates the p-4E-BP1 level in the hippocampus and amygdala regions of the brain	Depression	Neuropsychiatric	[199]
4E-BP1	Decreased levels of p-4E-BP1 were reversed by Alarin neuropeptide, which reported a potent antidepressant effect	Depression	Neuropsychiatric	[200]
4E-BP1	CACNA1C, which encodes for a Ca^+^ channel subunit, when knocked out in mice, showed decreased levels of mTORC1 and 4E-BP1 in the prefrontal cortex with severe anxiety and impaired social behavior	Depression	Neuropsychiatric	[202]
4E-BP1	A 5-HT1A receptor agonist increased phosphorylation levels of mTOR and 4E-BP1, indicating increased protein translation	Depression	Neuropsychiatric	[203]
4E-BP1	Haloperidol, an anti psychotic drug, activates phosphorylation of 4E-BP1	Schizophrenia	Neuropsychiatric	[210]
4E-BP2	4E-BP2 KO mouse reported a disbalance in synaptic function, leading to ASD like behavior	Autism Spectrum Disorders	Neurodevelopmental	[167]
4E-BP2	4E-BP2 KO in inhibitory neurons causes ASD traits in mice but not in excitatory neurons	Autism Spectrum Disorders	Neurodevelopmental	[94]
4E-BP2	4E-BP2 downregulation in cerebellar Purkinje cells interrupts the mTORC1, leading to ASD like behavior	Autism Spectrum Disorders	Neurodevelopmental	[93]
4E-BP2	4E-BP2 KO in inhibitory neurons lowered seizure threshold in mice	Epilepsy	Neurodevelopmental	[179]

## Neurodevelopmental and Neuropsychiatric Disorders and 4E-BP’s

### Autism Spectrum Disorder

Autism spectrum disorder (ASD) is a neurodevelopmental disorder characterized by repetitive behavior and difficulty in social interaction and communication. Males are at a higher risk of ASD than females. Disease may develop because of the interplay between multiple genetic and environmental factors, and heritability is estimated to be as high as 50–90% [162, 163]. It is usually diagnosed by behavioral observation by a professional. Treatment includes speech or social therapy and medications for symptoms such as anxiety [164].

The effects of downstream proteins of mTOR, such as 4E-BP2 and eIF4E, on neuroligins (cell adhesion proteins) located at the postsynaptic membrane have been estimated [165]. 4E-BP2 KO results in autistic-like behaviors in mice, including deficits in social interaction and communication [166]. The three-chamber social arena test and the social interaction test revealed that 4E-BP2-KO mice exhibited reduced social interaction behavior. These mice exhibited longer self-grooming and buried more marbles than did the WT, which indicates anxiety-like behavior. Isolation-induced ultrasonic vocalizations (USVs) emitted from these mice were greater than those emitted from WT mice, mirroring ASD traits. 4E-BP2 KO leads to increased charge transfer for miniature excitatory postsynaptic currents (mEPSCs) and miniature inhibitory postsynaptic currents (mIPSCs), causing an altered ratio of synaptic excitation to inhibition (E/I) balance, which was reversed by the knockdown of *Nlgn1,* an adhesin neuroligins (NLGNs)-neurexins which maintain E/I ratio*.* Knockdown of *Nlgn1* can also partially rescue ASD like symptoms. These findings suggest that 4E-BP2 and eIF4E play important roles in postsynaptic translational control in neurons [167].

4E-BP1 or eIF4E levels were unchanged in ASD patient samples, whereas other mTOR proteins, such as p70S6K and eIF4B, were decreased. Compared with control rats, valproic acid (VPA)-treated rats presented decreased levels of Akt, p-Akt, mTOR, p-mTOR, p-S6, 4E-BP1 and p-4E-BP1 [168]. 4E-BP2 was specifically deleted in excitatory and inhibitory interneurons as well as astrocytes to understand the cell-specific effect of 4E-BP2. 4E-BP2 KO in inhibitory neurons causes ASD traits in mice but not in excitatory neurons [94] (Fig. 6). 4E-BP2 KO in Purkinje cells revealed that 4E-BP2 primarily regulates memory rather than ASD behaviors. The mice exhibited memory deficits but no ASD traits [93].

### Epilepsy

We categorize epilepsy as a neurodevelopmental disorder because alterations in brain development during childhood contribute to its origin, along with shared genetic mechanisms and comorbidities with other neurodevelopmental conditions, including autism spectrum disorder and intellectual disability [169, 170]. Epilepsy is a neurological disorder characterized by recurrent seizures stemming from abnormal brain activity. These seizures can be mild to severe, with a range of symptoms, including involuntary movements, brief loss of awareness, or muscle rigidity. There are 2 types of seizures: generalized and focal. Focal seizures are caused by abnormal activity in one localized region of the brain [171–173]. There are many causes of focal seizures, such as structural abnormalities, genetic conditions, head trauma, and tumors. A few such seizures are temporal lobe seizures, frontal lobe seizures, occipital lobe seizures, and parietal lobe seizures. Generalized seizures are associated with abnormal electrical signals in both halves of the brain. There are 3 types of generalized seizures: absence seizures, myoclonic seizures, and generalized tonic–clonic seizures [174].

Epilepsy-associated glioneuronal tumor samples from patients presented no significant changes in p-4E-BP1 levels compared with those from controls [175]. Dysembryoplastic neuroepithelial tumors (DNTs) with p-S6 and p-4E-BP1 expression were immunopositive in 89.7% of cases in patients. This explains the role of mTOR as a therapeutic target in epilepsy [176]. Focal temporal lobe epilepsy (TLE) is characterized by an enlarged and dispersed granule cell layer in the dentate gyrus. Upregulated mTORC1 is observed in TLE patients, and the downregulation of mTORC1 was protective against seizures. The mice were induced to seizures with kainic acid (KA) and treated with eugenol (an anticonvulsant herb) and naringin (present in citrus), which delayed seizures and decreased granule cell dispersion (GCD). Mice injected with KA presented increased levels of p-4E-BP, whereas those treated with these phytopharmaceuticals presented reduced levels. These findings suggest that these phytochemicals act by reducing GCD through the inhibition of mTORC1 activation [177].

Epileptic mice suffering from focal malformations of cortical development (FMCD) were observed to have mutations in genes associated with the mTOR pathway. eIF4E inhibition by metformin rescued these mice from FMCD and seizures. This explains the role of eIF4E or its inhibitors, such as 4E-BP1, as therapeutic targets in epilepsy [178]. Mice with 4E-BP2 deletion in parvalbumin inhibitory interneurons were more susceptible to seizures when treated with 50–70 mg/kg pentylenetetrazole (PTZ) or 30 mg/kg KA. 4E-BP1, 4E-BP2 and 4E-BP3 triple-KO mice (eIF4E-BP^−/−/−^) treated with PTZ or KA presented a reduction in the seizure threshold and an increase in seizure-associated mortality. 4E-BP1 KO alone did not cause a reduction in symptoms, whereas 4E-BP2 KO mice presented a reduction in the seizure threshold, prolonged seizure duration, and increased mortality. There was no difference in seizure activity in excitatory neuron-specific conditional-KO mice, eIF4E-BP2^flx/flx^: Emx1-Cre^+^. However, a reduction in the seizure threshold was observed in inhibitory neuron-specific conditional-KO eIF4E-BP2^flx/flx^: Nkx2.1-Cre^+^ mice. This explains why 4E-BP2 influences inhibitory rather than excitatory neurons. To analyze seizures via electroencephalography, a bipolar recording electrode was implanted into the hippocampus. To subtype affected inhibitory neurons, highly specific inhibitory neuron subclass knockouts were generated for somatostatin (SST), vasoactive intestinal peptide (VIP), and parvalbumin (PVALB) neurons in eIF4E-BP 2^flx/flx^ mice. A decreased number of parvalbumin neurons in the hippocampus was observed in the 4E-BP2 PVALB conditional-KO mice. These results suggest that 4E-BP2 is the main target downstream of mTOR, which regulates epileptogenesis in specific neuronal subclasses [179].

FMCD-fed mice presented increased p-4E-BP1, which is indicative of increased protein translation. Through a constitutively active form of 4E-BP1 that resists phosphorylation, a reduction of focal malformation and reduced neuronal cytomegaly was observed. Different neuronal electrophysiological alterations, such as depolarized resting membrane potential, irregular firing patterns, and hyperpolarization-activated cyclic nucleotide-gated isoform 4 (HCN4) ion channels, were normalized. When 4E-BP1 expression was initiated after the onset of epilepsy, decreased seizures and improved overall activity in mice were observed [180]. Taken together, these findings indicate that increased protein translation might contribute to epilepsy. Logically, targeting 4E-BP1 or 4E-BP2 may be a potential therapeutic alternative [181].

### Guillain–Barré Syndrome

Guillain–Barré syndrome (GBS) results from an autoimmune response that targets peripheral nerves which is triggered by an infection. The immunogenic similarity between nerve components and certain microbes is the main cause, yet the precise details remain unknown [182, 183]. The primary symptoms include ascending weakness from the legs and motor coordination difficulty. Severe cases may lead to paralysis [184]. Plasmapheresis is used to remove autoantibodies from the blood, followed by intravenous immunoglobulin (IVIG), which binds to the autoantibodies and helps in managing the condition [185]. The protein levels of the AKT-mTOR pathway and key autophagy markers were tested in GBS patients. There were no significant protein level changes, and the 4E-BP1 levels were also normal. Therefore, the current data suggests that alterations in protein translation are not major contributors to GBS pathophysiology [186].

### Depression

Depression is one of the most common neuropsychiatric disorders. It is characterized by sadness, a lack of interest in activities, insomnia, and trouble concentrating [187, 188]. The major types of depression are: Major Depressive Disorder (an acute condition with severe symptoms); Persistent Depressive Disorder (a chronic condition with mild symptoms); Bipolar Depression (depression with severe mood fluctuations); Seasonal Affective Disorder; Postpartum Depression; and Psychotic Depression (depression and psychosis). Psychotherapy and antidepressants such as the Selective Serotonin Reuptake Inhibitor (SSRI) are used to treat depression [189].

In models of depression, the antidepressant effects of certain drugs, especially rapid-acting drugs such as ketamine, are significantly reduced when an mTOR inhibitor (e.g., rapamycin) is administered [190, 191]. These findings suggest that mTOR signaling plays a critical role in mediating the effects of these drugs. This finding explains the role of mTOR in the pathophysiology of depression. The prefrontal cortex, amygdala, anterior cingulate cortex, and hippocampus are the main areas of the brain involved in depression [192, 193]. Most studies on depression have shown that these areas are affected when individuals are treated with antidepressants and their agonists [194]. Elevated levels of synaptic proteins and an increase in synapse number were observed following ketamine treatment. Ketamine activates the mTOR pathway, followed by the activation of its downstream targets, 4E-BP1 and p70S6K [195]. This effect lasts only ~ 2 h after administration and at a low dose of 10 mg/kg. Many different antidepressants, such as imipramine, fluoxetine, and electroconvulsive seizure therapy, do not affect the mTOR pathway. Inhibition of the mTOR pathway blocked synaptogenesis and prevented the antidepressant effects of ketamine in depression models. They examined the effects of NR2B agonists (which activate N- methyl D aspartic acid receptors), which function similarly to ketamine. Ro25-6981, an NR2B agonist, was found to activate mTOR, 4E-BP1, and p70S6K [196]. The antidepressant LY341495 increased the levels of various proteins, such as mTOR, p70S6K, and 4E-BP1, in a manner similar to that of ketamine. It also increased the levels of upstream targets of mTOR, such as p-ERK. The effect of this drug was inhibited by the addition of rapamycin [197]. Primary hippocampal cultures from Sprague‒Dawley rats were treated with antidepressants to analyze the levels of mTOR pathway proteins. Treatment with antidepressants such as escitalopram, paroxetine, and tranylcypromine increased the levels of mTOR, 4E-BP1, and p70S6K, whereas treatment with fluoxetine, sertraline, and imipramine did not affect the mTOR pathway. Upon the addition of rapamycin, there was no effect of the antidepressants. These findings suggest that at least some antidepressants may exert their effects, in part, through the mTOR pathway [198]. Fluoxetine, an SSRI, increased the phosphorylation of 4E-BP1 only in the hippocampus and amygdala, concomitant with increased phosphorylation of mTOR [199]. Mild stress was induced in the mice, followed by administration of the neuropeptide antidepressant alarin. Stress reduced p-4E-BP1/4E-BP1 levels in mice, but treatment with alarin restored these levels in the cortex, hippocampus, hypothalamus, and olfactory bulb. Treating mice with rapamycin, an mTOR inhibitor, negated this effect of alarin. These findings suggest the involvement of the mTOR-4E-BP1 pathway in depression [200]. The CACNA1C gene encodes a subunit of Ca^2^⁺ channels. The integrity of this gene is crucial for maintaining neuronal function and has been strongly implicated in neuropsychiatric disorders [201]. Anxiety and impaired social behavior are key features of CACNA1C-KO mice. There was a reduction in the levels of mTORC1 and 4E-BP1 in the prefrontal cortex. These findings support the hypothesis that 4E-BP1 may have a role in neuropsychiatric disorders [202]. When 8-OH-DPAT, a 5-HT1A receptor agonist, was injected subcutaneously into mice, higher phosphorylation levels of mTOR and 4E-BP1 were observed, indicating increased protein translation [203]. The antidepressant rapastinel works through the ERK-mTOR pathway, which increases the phosphorylation of 4E-BP1 and p70S6K, leading to increased protein translation of brain-derived neurotrophic factor (BDNF) and the neuropeptide VGF (nonacronymic), which works through a feedback loop that helps in the rapid release of the antidepressant [204]. Rosiglitazone (RGZ), a type 2 diabetes mellitus medication, is known to have neuroprotective effects on dexamethasone-induced depression. Dexamethasone is an immunosuppressive corticosteroid used to treat different neurological and autoimmune conditions. When the mice were treated with 20 mg/kg Dexamethasone, elevated levels of mTOR and 4E-BP1 were observed. Whereas RGZ administration drastically decreased these levels. Higher levels of protein translation after Dexamethasone treatment may cause protein dysfunction in mice, which is rectified after RGZ administration. RGZ acts as a neuroprotective agent by inhibiting the AKT-MAPK-mTOR pathway and increasing nerve growth factor [205].

### Schizophrenia

The neuropsychiatric disorder schizophrenia is characterized by changes in cognition and emotional regulation, hallucinations, delusions, and disorganized speech and thinking. The mechanisms underlying this disorder have not been fully explored. MK-801 is usually used to model schizophrenia-like symptoms in rodents. This resulted in alterations in mTOR signaling (increased phosphorylation of Akt, 4E-BP1, and p70S6K), which may contribute to synaptic dysfunction and behavioral abnormalities [206–209]. These results suggest the involvement of the mTOR pathway in schizophrenia. Primary neurons treated with the antipsychotic haloperidol presented increased levels of phosphorylated mTOR, S6, and 4E-BP1. There was increased striatal neuron branching upon haloperidol treatment. When 4E-BP1 was inhibited, protein synthesis and neuron branching decreased even during treatment with haloperidol [210, 211]. The whole blood of treatment-resistant schizophrenia (TRS) patients was compared with that of healthy controls. The results revealed increased mTOR, P70S6K, and 4E-BP1 levels, but only P70S6K reached statistical significance. These studies highlight a potential role for mTOR signaling in schizophrenia; however, further research is needed to determine how best to target 4E-BP1 and mTOR for therapeutic intervention [212].

### Multiple Sclerosis

Multiple sclerosis is an autoimmune condition that affects the central nervous system. T cells and B cells attack the myelin sheath around axons, leading to demyelination, neuroinflammation, and axonal damage. This disorder is characterized by neurological issues such as numbness and tingling cognitive issues such as loss of memory and difficulty concentrating, sensory issues such as poor vision, and motor issues such as difficulty in walking and coordination [213, 214]. There are four different types of multiple sclerosis (MS):

(i) Primary progressive MS (PPMS): Gradually worsens without periods of remission.
(ii) Secondary progressive MS (SPMS): This disease initially follows a relapsing‒remitting course but later progresses steadily, with or without remission.
(iii) Relapsing–Remitting MS (RRMS): It is characterized by alternating periods of symptom flare-ups (relapses) and recovery (remission).
(iv) Progressive-Relapsing MS (PRMS): A progressive form of the disease with intermittent periods of worsening symptoms (relapses).

There is no cure, but treatments to manage different symptoms are usually prescribed [215]. The mRNA expression levels of mTOR, RPS6KB1, and 4E-BP1 were analyzed in the blood of MS patients and healthy controls. There was a significant increase in the levels of mTOR, RPS6KB1, and 4E-BP1 in MS patients compared with those in controls. This study highlights a potential role for the mTOR pathway in MS pathophysiology. However, the exact mechanisms by which this occurs remain unclear [216, 217]

## Effects of 4E-BP’s on Fetal Development and the Central Nervous System

### Fetal Development

Spn-2 is the homolog of 4E-BP1 identified in *Caenorhabditis elegans.* Spn-2 is an 84 kDa protein that localizes to the cytoplasm and regulates MEI-1 during embryonic development in *C. elegans.* Spn-2 binds with OMA-1, which later binds to MEI-1 and regulates translation. MEI-1 is a part of the enzyme complex of katanin microtubules. Its activity is required for the formation of a meiotic spindle during various developmental stages, but its activity must be downregulated before embryogenesis to prevent the formation of mitotic spindle defects. Sequence similarity and binding assays were used to characterize spn-2 as a 4E-BP1 homolog. However, spn-2 lacks the complete eIF4E binding structure of human 4E-BP1, but its N-terminal domain is conserved, which proves sufficient for its binding with eIF4E [218]. 4E-BP1 plays an important role in the growth of *Bombyx mori* eggs. Some silkworm embryos undergo diapause, which inhibits their growth; this diapause can be reversed by HCl treatment after oviposition (the process of laying eggs). The molecular mechanism by which diapause is rescued remains unknown. Interestingly, following HCl treatment, the authors noted a drastic surge in 4E-BP1 phosphorylation, leading to increased protein translation. This was maintained throughout the worm stage of development. When eggs were treated with LY294002 and rapamycin, a decrease in p-4E-BP1 levels was observed, potentially explaining the involvement of the mTOR and PI3K mechanisms in development [219]. Several molecules can increase spermatogenesis and the number of spermatogonial stem cells. CHIR99021, Kenpaullone, Pifithrin-α, Dorsomorphin, SB431542, A83-01, and PD0325901 were added to isolated spermatogonia from human testes. These drugs have also been tested in mice to understand their effects on spermatogenesis in humans and mice. Spermatogonia proliferation increased in isolated spermatogonia from patients and in mice treated with SB431542. 4E-BP1 was also significantly downregulated with SB431542 treatment. Through qRT‒PCR quantification, they reported that the TGFb/Smad2/3 pathway was likely responsible for the decrease in the expression of cyclin-dependent kinases and increase in the expression of the 4E-BP1 and p57 genes, which was reversed by the addition of an SB431542 inhibitor [220]. mTOR plays an important role in regulating fetal brain development in Suffolk sheep with prenatal alcohol exposure. They found that p-S6K was upregulated, whereas p-4E-BP1 was not significantly changed in alcohol treated ovines compared to the control. Glutamine supplemented the alcohol exposure paradigm, which caused mTOR phosphorylation to increase in the fetal brain. These results suggest the involvement of mTOR in fetal alcohol syndrome (FAS), but the detailed mechanism is yet to be understood [221]. Rat fetuses also showed drastic increases in the levels of total and p-4E-BP1 and a decrease in p-mTOR in the hippocampus with alcohol exposure. These studies highlight the need to explore the mTOR pathway in detail in the context of the effects of chronic alcoholism on fetal development [222–224].

### Brain Function

Zinc is essential for axonal development, and its relationship with the mTOR pathway is unexplored. Primary hippocampal neurons isolated from the rat brain were used to analyze the role of zinc and characterize the phosphorylation status of mTORC1 and 4E-BP1. One key observation was that an increase in axon length was correlated with the 4E-BP1 phosphorylation level [225]. In neurons, the soma is where protein translation occurs, but reports have demonstrated that protein synthesis operations are confined to dendrites. The RNA granules in dendrites contain most of the machinery required for protein synthesis but lack a few important initiation factors. eIF4E is one missing factor; it is typically present in the postsynaptic space, but it can be shifted to dendrites when needed. 4E-BP1 mRNA was found throughout the length of the neuron, with the highest levels in the cell body (soma). The 4E-BP1 protein present in dendrites can be upregulated by neuronal activation in response to treatment with 60 mM KCl for 10 min. Except for 4E-BP1, most initiation factors are enriched in the subsynaptic space, facilitating local protein synthesis at dendritic spines. Two reasons that such local translation is needed are synaptic plasticity and rapid and localized responses to activity [226]. Rheb overexpression in primary and embryonic neurons in mice has a prime role in axonal elongation. The inactive form of Rheb reduces mTORC1 activity, leading to decreased 4E-BP1 phosphorylation. However, under these conditions, 4E-BP1 is phosphorylated through mTORC1-independent mechanisms. The cells were treated with the mTOR inhibitor rapamycin, and no change in axonal length was observed, suggesting that mTOR may not play a major role in regulating axonal length under these conditions. However, further genetic experiments are required to validate these results. The exact mechanisms by which Rheb promotes 4E-BP1 phosphorylation need to be further studied [227].

### Spinal Cord Function

To understand the role of the mTOR pathway in the transmission of pain, the levels of mTOR, 4E-BP1, and p70S6K were analyzed in the dorsal root ganglion (DRG) and spinal cord. All three proteins were expressed, whereas their phospho forms presented minimal to no expression. First, in the DRG, mTOR and p70S6K were expressed in neurons, whereas 4E-BP1 was found in satellite glial cells. In contrast, in the dorsal horn of the spinal cord, all three proteins were detected in neurons but not in glial cells. Rapamycin treatment did not affect pain transmission in the rat models, suggesting that the mTOR pathway may not be involved in pain regulation [228–231].

## Other Brain-Related Disorders

The anesthetic tiletamine caused increased phosphorylation of LKB1 and AMPK, whereas the levels of 4E-BP1 were decreased in the cerebral cortex, hippocampus, thalamus, cerebellum, and brainstem in the rat model. The activation of AMPK inhibits the activity of mTOR and, in turn, reduces 4E-BP1 phosphorylation [232]. Stroke and ischemic postconditioning (IPostC) is an alteration between ischemia and reperfusion in the brain after stroke. One study analyzed different proteins from important signaling pathways and correlated them with different immune cell types in IPostC. Mass cytometry revealed that the level of p-4E-BP1 is increased in CD4 + T cells, CD8 + T cells, and classical dendritic cells (cDCs) and decreased in microglia and monocyte-derived macrophages (MoDMs), explaining its importance in protection by IPostC [233].

## Therapeutic Strategies

Therapeutic strategies are necessary for different neurological disorders. There are no direct inhibitors against 4E-BPs. However, mTOR or mTORC1 inhibitors can inhibit the phosphorylation of 4E-BPs and regulate protein translation. Many different inhibitors have been identified and tested in different cancers and other diseases, along with neurological disorders. Given that many compounds have not been widely studied from a neurological perspective, we aimed to establish the therapeutic potential of these compounds in targeting the mTOR-4E-BP1 axis [234–236]. Many of these inhibitors are in different clinical trials for different disorders **(**Table 3**)**. mTOR inhibitors are broadly classified into the following types [234, 235].

**Table 3:** Summary of several major clinical trials of different inhibitors targeting essential proteins in the mTOR pathway. The data were obtained from https://clinicaltrials.gov

Inhibitors	Condition	Clinical trial
Rapamycin	Hysteromyoma	Phase IV
	Squamous cell skin cancer	Phase II
	Solid tumor	Phase I
	Alzheimer disease	Phase I/II
	ALS	Phase II
Temsirolimus	Non-Hodgkin’s lymphoma	Phase IV
	Hepatoblastoma	Phase III
	Breast cancer	Phase II
	Glioblastoma	Phase I/II
Everolimus	Autism and Neuro Psychological Deficits	Phase II/III
	Low-Grade Glioma	Phase II
	Prostate Cancer	Phase I
	Atypical Hyperplasia or Stage IA Grade 1 Endometrial Cancer	Phase II
	Metastatic or Unresectable Kidney Cancer	Phase II
	Subependymal Giant Cell Astrocytoma	Phase I/II
	Epilepsy	Phase I/II
Ridaforolimus	Soft tissue and bone sarcomas	Phase III
	Endometrial cancer	Phase II
WRX606	It showed tumor suppression in the xenograft mouse models	-
Torin 1	It has been studied widely in preclinical models of colon cancer	-
Torin 2	It has been studied widely in preclinical models of hepatocellular carcinoma	-
MLN0128	Advanced Nonhematologic Malignancies	Phase I
	Prostate cancer	Phase II
Rapalink −1	It showed tumor suppression in preclinical models of sarcoma, prostate cancer, and renal cell carcinoma	-
Dactolisib	It showed decreased neuronal death and memory impairment in preclinical models of AD	-
Omipalisib	Solid tumors	Phase I
Bimiralisib	Head and neck squamous cell carcinoma	Phase II
	Breast cancer	Phase II
Gedatolisib	Breast cancer	Phase I/II
Voxtalisib	Relapsed/refractory lymphoma	Phase II
Apitolisib	Metastatic castration-resistant prostate cancer	Phase II
	Advanced solid tumors	Phase I
	Metastatic renal cell carcinoma	Phase II
DI06	It showed tumor suppression in preclinical models of hepatocellular carcinoma models	-
4EGI-1	It binds to eIF4E and induces a conformational change that dissociates its interaction with eIF4G	-
4E1RCat	It binds to eIF4E and physically inhibits its association with eIF4G and 4E-BP1	-
Ribavirin	Acute Myeloid Leukemia	Phase II
	Hepatitis C	Phase II
Rifabutin	TB-HIV coinfection	Phase I/II
EGPI-1	It showed tumor suppression in preclinical models of lung cancer	-

### Allosteric Inhibitors

These contain Rapalogs and Non-Rapalogs.

- *Rapalogs*: Rapamycin was extracted from *Streptomyces hygroscopicus*. Rapamycin binds to the FRB domain of mTOR and blocks it. This effect affects the function of mTOR as a kinase, further inhibiting mTORC1 and mTORC2 complexes. These Rapalogs promote autophagy and clear toxic proteins such as tau and amyloid β. These rapalogs neutralize the hyperactive mTOR pathway in several neurodevelopmental disorders, such as epilepsy [141, 237–239]. There are many variants, such as temsirolimus, everolimus, and ridaforolimus, which have the same backbone structure but differ at the C42 position. Temsirolimus is used as a treatment against AD and PD, whereas everolimus is used against AD, neuroinflammation and breast cancer, and ridaforolimus is used in osteosarcoma [240–243].
- Nonrapalogs: A nonrapalog inhibitor, WRX606, was developed against mTOR, which allosterically binds and inhibits its function. It has been shown to significantly reduce tumor progression in animal models. These inhibitors affect mTORC1 and the phosphorylation of S6K and 4E-BP1 but affect mTORC2 only upon longer exposure. These inhibitors cannot completely inhibit mTORC1 and weakly inhibit the mTORC2 complex [238, 242, 244].

### ATP Competitive mTOR Inhibitors

These inhibitors target the ATP-binding segment of mTOR and block both mTORC1 and mTORC2. This causes full-scale inhibition of mTOR activity, which in turn suppresses S6K and 4E-BP1. These inhibitors can be divided into several subtypes based on their structure [239].

- *Morpholine inhibitors*: These compounds contain a morpholine moiety, which is similar to adenosine in ATP and helps the inhibitor bind to the mTOR ATP binding pocket. Approximately 30 compounds with a morpholine core heterocycle backbone have been identified. Although the majority of these compounds significantly inhibited mTOR in vitro, they could not effectively pass all the pharmacological assessments in vivo. Although these two compounds entered clinical trials, they were subsequently withdrawn [245, 246].
- *Quinoline inhibitors*: Torin 1 has a quinoline backbone and can significantly inhibit mTOR activity. It has a very short half-life along with poor solubility. Torin 2 does not contain a propionylpiperazine group, and the quinoline is replaced by aminopyridine. It shows similar mTOR inhibition and has better solubility than Torin 1. Torin has very minimal permeability through the blood–brain barrier, limiting its use in neurological disorders [247–249].
- *Pyrazolo (3,4-d) pyrimidin 4 amine inhibitors*: MLN0128 (Sapanisertib) binds to the ATP-binding domain of mTOR. It can inhibit mTORC1 and mTORC2 better than rapalogs. This further inhibited the phosphorylation of S6K and 4E-BP1. MLN0128 treatment reduces tumor formation in various types of cancer. It is in phase I and II clinical trials against different cancers, such as prostate cancer. It is known to cause toxicity in many patients during the course of a trial, and reducing the dosage causes incomplete target inhibition [250–254].

### Multiple Binding Site Inhibitors

Rapalink-1 was developed by connecting rapamycin with MLN0128 with a linker chain sequence. This compound can target the rapamycin binding site along with the ATP binding site of mTOR. It also reduced the phosphorylation of 4E-BP1. This compound has shown stronger inhibition along with better tumor suppression without being significantly toxic in *in vitro* and *in vivo* models of different types of cancer, such as sarcoma, prostate cancer, and renal cell carcinoma. Although clinical trials are needed to fully understand this compound, this work has opened a new era of mTOR inhibitors [255–259].

### Inhibitors Targeting Multiple Pathways

- *mTOR and PI3K inhibitors*: These inhibitors target the structurally similar p110 subunits of both proteins. These inhibitors have been shown to have better tumor suppression properties against different cancers. Dactolisib [260–262], omipalisib [263], bimiralisib [264], gedatolisib [265], voxtalisib [266] and apitolisib [267] are in Phase I and II clinical trials. Dactolisib has been shown to decrease neuronal death and memory impairment in AD models [261, 262].
- *mTOR and HDAC inhibitors*: Although targeting HDACs to treat cancers has been established, targeting both mTOR and HDACs has been shown to have promising results. Among them, 12 l pyrimidine‒pyrazolyl pharmacophore is known to inhibit mTOR and stimulate apoptosis in hematological malignancies. Another study connected MLN0128 and the HDAC inhibitor SAHA (vorinostat) and created a hybrid called DI06, which suppressed tumors in hepatocellular carcinoma models. Further studies are needed on mTOR-HDAC inhibitors to further understand their potential [268, 269].
- *mTOR and autophagy inhibitors*: This combination increases autophagic flux, clears protein aggregates, reduces neuronal stress, and restores cellular homeostasis. The combination of rapamycin and trehalose increased autophagy, improved neuronal recovery, and promoted neuroprotection in MPTP-induced mouse models. When rapamycin is used in combination with spermidine, it reduces toxic aggregation and accumulation of amyloid β plaques and helps improve cognitive function. MLN0128, used in combination with autophagy inducers, resulted in reduced tau and amyloid β aggregation in AD [270–273].

### 4E-BP and eIF4E Interaction Disruptors

- *4EGI-1*: It binds to eIF4E and induces a conformational change that dissociates its interaction with eIF4G. It also promotes the binding of 4E-BP1 to eIF4E. These 4EGI-1 inhibit protein translation. Further research is needed to understand the potential of 4EGI-1 in neurodegenerative disorders [274–276].
- *4E1RCat*: It binds to eIF4E and physically inhibits its association with eIF4G and 4E-BP1. It is known to inhibit protein translation [277, 278].
- *Ribavirin*: is an antiviral compound that binds to eIF4E and diminishes its binding to the m7G cap of mRNA. It reduces cancer cell proliferation in lymphoblastic leukemia, breast cancer, and other solid tumors [279–282].
- *Rifabutin*: It is an antibiotic that targets eIF4E and inhibits its phosphorylation at S209. It has shown significant potential in the treatment of lung cancer [283, 284].
- *EGPI-1*: It binds to eIF4E, inhibits its interaction with eIF4G, and inhibits the phosphorylation of 4E-BP1. It has shown significant potential in the treatment of lung cancer [236, 285].

## Limitations and Prospects of mTOR Inhibitors

mTOR regulates diverse functions of the cell, such as glucose, lipid, and protein metabolism, mitochondrial functions cytoskeleton organization, and ion transport. In turn, it is connected to other signaling pathways, such as the MAPK/ERK, PI3K/AKT, AMPK, and Wnt signaling pathway, hypoxia and autophagy. These findings suggest that mTOR is a key player in neurological disorders, metabolic disorders, cancer, and even immunological disorders. Targeting mTOR can surely regulate the mechanism associated with many disorders, but the off-target effects may outweigh the benefits, which is its major limitation. This has proven to be detrimental during the clinical trials of many inhibitors, leading to discontinuation of the study. Many of these inhibitors do not cross the blood‒brain barrier, limiting their use in neurological disorders.

Allosteric inhibitors targeting mTOR primarily target mTORC1 and partially inhibit mTORC2. They activate a negative feedback loop, which also contributes to their weak inhibition. They are difficult to synthesize because of their complex structure. The long-term use of these drugs is not recommended because of significant side effects. Although ATP competitive inhibitors target both mTORC1 and mTORC2, they exhibit side effects. This is possibly due to the inhibition of almost all the pathways regulated by mTOR. Although combinatorial inhibitors target more than one pathway and have good inhibitory effects, they exhibit toxicity. Unknown pathway cross-talk may occur, which might be a significant limitation. 4E-BP/eIF4E complex disruptors are a better choice for regulating downstream pathways, such as protein translation, as other pathways regulated by mTOR are not disrupted. These compounds have low off-target effects and low toxicity.

Thus, targeting downstream molecules of mTOR could solve the problem of toxicity with minimal side effects. One of the significant areas to be pursued is identifying inhibitors that target 4E-BPs directly instead of mTORC1. Another unexplored area is the testing of these existing inhibitors in neurological disorders such as neurodegeneration, neurodevelopmental, and neuropsychiatric conditions. Novel drug development strategies are required to develop inhibitors with improved blood‒brain barrier permeability that can significantly regulate protein translation without altering other interconnected pathways [234–236].

## Conclusion

The eukaryotic translation initiation factors 4E-BP1, 4E-BP2, and 4E-BP3 serve as critical regulators of cap-dependent protein synthesis, directly influencing neuronal function and pathology. While all three isoforms play a common role in inhibiting eIF4E-mediated translation, their distinct expression patterns and regulatory mechanisms suggest specialized functions in the nervous system. 4E-BP2 is the major isoform expressed in the brain and has been closely linked to synaptic plasticity and cognitive function, whereas 4E-BP1 and 4E-BP3 contribute to broader stress responses and neuroprotection. 4E-BP3 compensates for 4E-BP1 and 4E-BP2 under sustained inhibition of mTOR.

Hyperphosphorylation of 4E-BPs by different kinases leads to hyperactive protein translation, causing protein aggregation. This dysregulation of 4E-BPs is increasingly recognized as a contributing factor in various neuronal disorders, including neurodegenerative diseases, epilepsy, and neurodevelopmental conditions. For example, 4E-BP2 dysregulation in inhibitory neurons leads to ASD, and 4E-BP1 dysregulation in dopaminergic neurons leads to PD. Aberrant mTORC1 signaling, a key regulator of 4E-BP activity, has been implicated in these pathologies, highlighting the therapeutic potential of targeting 4E-BPs to restore translational homeostasis. However, selectively modulating 4E-BP function without disrupting essential cellular processes remains a major challenge.

Future research should aim to unravel the isoform-specific roles of 4E-BPs in neuronal health and disease, explore their interactions with other translational regulators, and identify strategies to fine-tune their activity. A significant amount of studies are needed to understand the specific role of 4E-BP3 and its regulation. In addition, studying all homologs of the 4E-BPs individually in different neurological disorders will help elucidate their unique role. Many mTOR inhibitors are not studied exclusively from a neurological perspective, as many cannot cross the blood‒brain barrier. Therefore, identifying specific inhibitors that cross the blood‒brain barrier will advance this area of research. Studying multiple inhibitors as combination therapies for neurological disorders, which are widely studied in cancer, may reveal new ways to regulate protein translation. There is a need to target downstream targets of mTOR, such as 4E-BPs, eIF4E, and eIF4G, instead of targeting mTORC1 or mTORC2 directly due to significant side effects. There are no known inhibitors targeting the 4E-BPs directly. A deeper understanding of these mechanisms could pave the way for novel therapeutic approaches aimed at restoring proper translational control in neurological disorders.

## Data Availability

No datasets were generated or analysed during the current study.

## Abbreviations

eIF4E-BP1: Eukaryotic translation initiation factor 4E-binding protein 1
eIF4E-BP2: Eukaryotic translation initiation factor 4E-binding protein 2
eIF4E-BP3: Eukaryotic translation initiation factor 4E-binding protein 3
PHAS-I: Phosphorylated heat and acid-stable protein regulated by insulin I
mTOR: Mechanistic target of rapamycin
FRAP: FKBP12 rapamycin-associated protein
SEP: Sirolimus effector protein
RAPT1: Rapamycin target 1
RAFT1: Rapamycin and FKBP12 target
mTORC1: Mechanistic target of rapamycin complex 1
mTORC2: Mechanistic target of rapamycin complex 2
PRAS40: Proline-rich Akt substrate of 40 kDa
Rictor: Rapamycin-insensitive companion of mammalian target of rapamycin
Raptor: Regulatory associated protein of mTOR
mLST8: Mammalian lethal with Sec13 protein 8
Deptor: DEP domain containing mTOR-interacting protein
eIF4E: Eukaryotic translation initiation factor 4E
eIF1: Eukaryotic translation initiation factor 1
eIF1A: Eukaryotic translation initiation factor 1A
eIF2: Eukaryotic translation initiation factor 2
eIF3: Eukaryotic translation initiation factor 3
eIF3J: Eukaryotic translation initiation factor 3 J
eIF4A: Eukaryotic translation initiation factor 4A
eIF4B: Eukaryotic translation initiation factor 4B
eIF4F: Eukaryotic translation initiation factor 4F
eIF4G: Eukaryotic translation initiation factor 4G
eIF4H: Eukaryotic translation initiation factor 4H
eIF5: Eukaryotic translation initiation factor 5
eIF5B: Eukaryotic translation initiation factor 5B
eRF1: Eukaryotic release factor 1
miRNA: MicroRNA
IGF1: Insulin-like growth factor 1
Rho-GDI: Rho guanine nucleotide dissociation inhibitor
HSC70: Heat shock cognate protein 70
NDKA: Nucleoside diphosphate kinase A
DRP2: Dystrophin-related protein 2
UCHL1: Ubiquitin C-terminal hydrolase L1
ADK1: Adenylate kinase 1
SOD1: Superoxide dismutase 1
Mef2c: Myocyte enhancer factor 2C
TFE3: Transcription factor E3
APP: Amyloid protein precursor
PSEN1: Presenilin-1
PSEN2: Presenilin-2
PI3K: Phosphatidylinositol 3-kinase
Akt: RAC-alpha serine/threonine-protein kinase or Protein Kinase B
MAPK/ERK: Mitogen activated protein kinase/ extracellular signal-regulated kinase
AMPK: AMP activated protein kinase
IGF-1: Insulin-like growth factor 1
ATP: Adenosine triphosphate
GTP: Guanosine triphosphate
CLOCK: Circadian locomotor output cycles protein kaput
BMAL1: Brain and muscle ARNT-like 1
SCN: Suprachiasmatic nucleus
VIP: Vasoactive intestinal peptide
Aβ: Amyloid beta
LRRK2: Leucine-Rich Repeat Serine/Threonine-Protein Kinase 2
PINK1: PTEN Induced Putative Kinase 1
PARK7: Parkinsonism-associated deglycase
MPTP: 1-Methyl-4-phenyl-1,2,3,6-tetrahydropyridine
IRES: Internal Ribosome Entry Site
IL-1β: Interleukin-1 beta
IL-6: Interleukin 6
TNF-α: Tumor necrosis factor alpha
cDCs: Classical dendritic cells
mEPSCs: Miniature excitatory postsynaptic currents
mIPSCs: Miniature inhibitory postsynaptic currents
BDNF: Brain derived neurotrophic factor
HCN4: Hyperpolarization activated cyclic nucleotide gated isoform 4
NFκB: Nuclear factor kappa B
Nrf2: Nuclear factor Erythroid 2-related Factor 2
